# Glioblastoma invasion into different organoid hosts reveals cell-intrinsic and proliferative migratory programs

**DOI:** 10.1016/j.isci.2026.115361

**Published:** 2026-03-13

**Authors:** Christopher Y. Akhunbay-Fudge, Bronwyn K. Irving, Alima Ismail, Sabrina Samuel, Emma Smedley, Holly E. Bradford, Steven Bagley, Alexander Baker, Iain M. Hagan, Deena M.A. Gendoo, Kevin Critchley, Ryan K. Mathew, Heiko Wurdak

**Affiliations:** 1 Stem Cells and Brain Tumour Group, Leeds Institute of Medical Research, School of Medicine, University of Leeds, Leeds LS9 7TF, UK; 2 Cancer Research UK Manchester Institute, The University of Manchester, Wilmslow Road, Manchester M20 4BX, UK; 3 Department of Cancer and Genomic Sciences, School of Medical Sciences, College of Medicine and Health, University of Birmingham, Birmingham B15 2TT, UK; 4 Institute for Data and AI, University of Birmingham, Birmingham, UK; 5 School of Physics and Astronomy, University of Leeds, Leeds LS2 9JT, UK; 6 Department of Neurosurgery, Leeds General Infirmary, Leeds LS1 3EX, UK

**Keywords:** Cell biology, Cancer

## Abstract

Akhunbay-Fudge et al. develop two complementary single-cell profiling methods to determine glioblastoma (GB) invasion phenotypes, focusing on the influence of host organoid developmental lineage (neural versus endodermal) and cell cycle progression on GB invasion within tumor assembloids. Notably, GB cells invaded both neural and endodermal organoid hosts, whereas non-malignant adult brain cells lacked this capacity. Single-cell mRNA sequencing revealed gene expression changes in invading tumor cells and surrounding environmental assembloid cells. Concurrently, the “DyPheT” automated tracking tool enabled real-time correlation of cell cycle phases with malignant cell migration within cerebral organoids, which can be utilized for treatment response assessment, exemplified by the investigational compound RP-6306. Collectively, these approaches identify an intrinsic (cell autonomous) gene expression signature linked to GB invasion and support a “go-and-grow” paradigm by revealing a highly migratory (and RP-6306-refractory) GB subpopulation active in the G2/M phase of cell cycle.

## Highlights

• GB cells invade neural and endodermal organoids showing lineage-independent infiltration
• Neural vs. endodermal assembloid comparisons reveal a cell-intrinsic GB invasion signature
• DyPheT enables automated tracking of GB cell cycle states and “go and/or grow” decisions
• DyPheT identifies a highly migratory G2/M phase GB subpopulation refractory to RP-6306

## Graphical abstract

**Figure:**
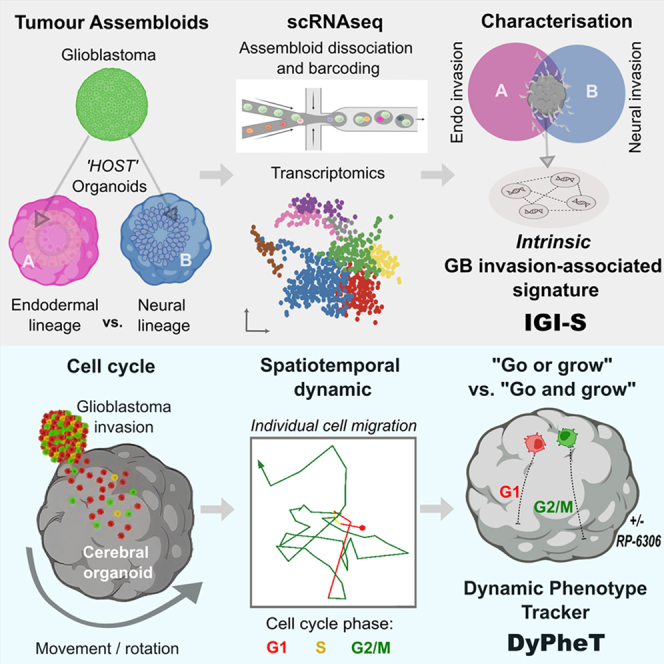

## Introduction

In a visionary study from 1938, Scherer concluded that brain tumor invasion is a process deeply intertwined with the interactions between tumor cells and the “pre-existing tissue” of the neural microenvironment.^1^ Despite decades of research and an increasingly neurocentric perspective on brain tumor biology,^2^^,^^3^ the cell-extrinsic (niche) and -intrinsic (cell autonomous) mechanisms driving malignant cell infiltration into healthy brain tissue remain insufficiently understood. Currently, there is no effective treatment that prevents the process of brain tumor invasion,^4^ making therapeutic intervention aimed at maximally eliminating infiltrative brain cancers and delaying tumor recurrence extremely challenging.

One prominent example is the most commonly occurring primary brain tumor, glioblastoma (GB), which is associated with a 5-year survival rate of <7% despite surgical debulking and adjuvant chemoradiotherapy.^5^ To some extent, these persistently poor outcomes can be attributed to the lack of *ex vivo* models that can mimic the biological complexity of GB while retaining compatibility for drug discovery and target validation approaches. In this context, patient-derived GB models maintained in stem cell-like phenotypes (either adherent^6^ or as tumor spheres^7^) have advanced the field by more accurately mimicking GB biology,^8^ providing an accessible cellular tool for identifying stemness,^9^ migration,^10^ and malignant cellular networks.^11^^,^^12^ The initiation of the organoid field,^13^ combined with the discovery of cerebral organoid (CO)-generating methods using embryonic and induced pluripotent stem cells (iPSCs),^14^^,^^15^^,^^16^ enabled methodologies that allow patient-derived GB (stem-like) cells to interact with, and infiltrate, neural tissue “hosts,” thereby forming 3D brain tumor models—so termed assembloids— in real time.^17^^,^^18^^,^^19^ Increasing evidence suggests that assembloid co-cultures of human GB cells and brain organoids better recapitulate phenotypic and molecular aspects of GB invasion, particularly when coupled with immunohistology and microscopy readouts and single-cell transcriptomics.^19^^,^^20^^,^^21^^,^^22^

In this study, we sought to investigate whether GB infiltration and invasive behavior depend on the host environment or can emerge independently of cellular context, and whether (and, if so, how often) invasive migration occurs in conjunction with cell division. Accordingly, our experimental approach focused on tumor-organoid assembloid models, using patient-derived GB cells and human iPSC-derived organoids that served as *in vitro* tumor modeling “host” tissues, to test whether GB invasion is modulated by the lineage identity of the specific assembloid environment. We generated early-stage neural and endodermal (foregut/hepatic-directed) host organoids, both selected for their coordinated differentiation timelines and distinct developmental lineage identities. Of note, the endodermal lineage gives rise to organs such as the liver and lung, which have been reported as rare sites of extracranial GB metastasis,^23^^,^^24^^,^^25^ hence offering a proof-of-concept approach for GB modeling beyond brain-mimicking organoid and tissue environments.

To identify host environment-dependent as well as intrinsic (cell autonomous and environment independent) GB invasion programs, we performed single-cell RNA sequencing (scRNA-seq) on dissociated neural and endodermal GB assembloids. This approach enabled systematic comparison of the transcriptional profiles across assembloids, revealing gene expression differences in individual tumor as well as environmental host cells. In parallel, we combined fluorescent cell cycle reporting with confocal microscopy-based live imaging of GB spheroids invading COs and developed the dynamic phenotype tracker (DyPheT), a tool for automated image analysis of individual GB cell trajectories. DyPheT enabled quantification of the migration behavior in relation to cell cycle phase oscillation, as well as the evaluation of chemically induced cell cycle perturbations. This allowed us to test whether GB invasion follows the “go-or-grow” and/or “go-and-grow” paradigms, providing insight into the extent of intertwined migration and proliferation in GB progression.^26^^,^^27^

Our complementary cell lineage modulation, cell profiling, and tracking methods reveal GB-induced changes in human iPSC-derived host environments and identify a cell-intrinsic gene expression signature of GB invasion. DyPheT analysis further identifies a “go-and-grow” invasion paradigm, where a subpopulation of highly migratory cells is active during cell division, rather than interphase. Accordingly, GB assembloids are amenable to methodological refinement, enabling advanced longitudinal observation of patient-derived GB cells *ex vivo*. These approaches can drive advancements in target identification, drug discovery, and aid in the development of anti-GB therapeutic strategies.

## Results

### Endodermal and neural lineage GB assembloids

**Figure 1:**
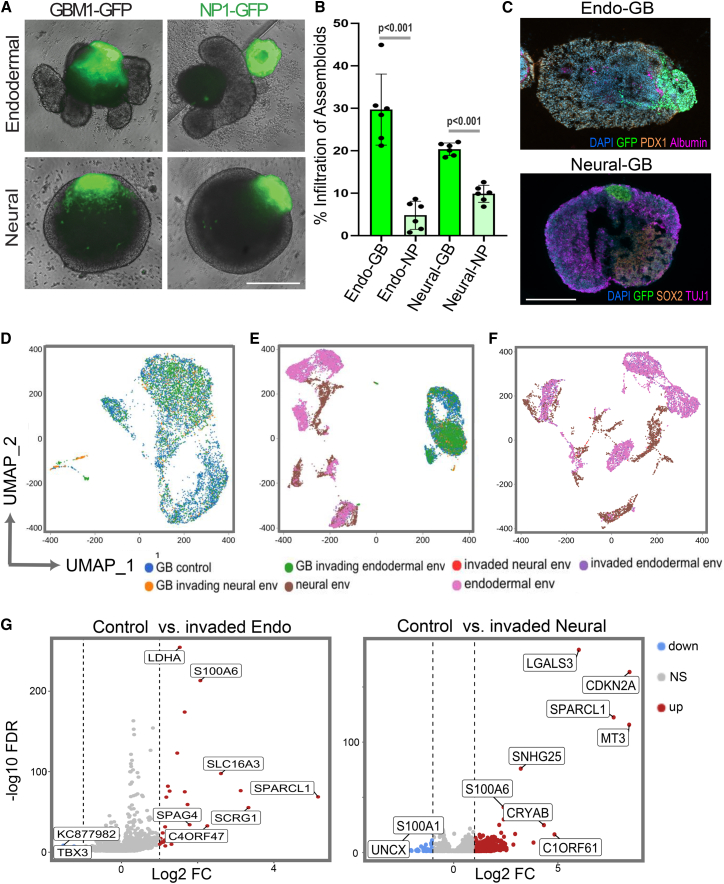
Generation and single-cell analysis of endodermal and neural lineage GB assembloids (A) GBM1 spheroids co-cultured with endodermal and neural lineage organoids for 48 h give rise to GB assembloids characterized by tumor cell invasion, in contrast to non-cancerous NP1 controls that exhibit organoid “docking” with limited infiltration. Scale bars: 300 μm. (B) Image-based quantification of GBM1 versus NP1 percentage infiltration into the indicated assembloids. Data are represented as individual values (dots) and mean (bar); error bars represent standard deviations from the mean. Statistical comparisons were performed using unpaired two-tailed Student’s *t* tests. (C) Cryosections of endodermal (endo-GB) and neural lineage (neural-GB) assembloids containing GFP-expressing GBM1 cells stained for the indicated markers. Scale bars: 250 μm. (D) UMAP of single-cell transcriptomes from pre-assembly (control) and assembloid-infiltrating GB cells at 72 h. (E) UMAP of all cells from GB assembloids, showing GFP-labelled GBM1 cells and invaded/non-invaded endodermal and neural lineage environment (env) cells. (F) UMAP highlighting invaded versus non-invaded environment cells from neural and endodermal lineage types. (G) Differential gene expression in invaded versus control host environments. Volcano plots show significantly upregulated (red), downregulated (blue), and non-significant (gray) genes (FDR < 0.01; log_2_ FC > 1 or < −1), with selected invasion-associated genes labeled. UMAP, Uniform Manifold Approximation and Projection. See also Figure S1.

To establish GB assembloids enabling different cell type compositions within host organoids for tumor cell invasion studies, we developed a throughput-compatible 14-day protocol. iPSCs were directed toward neural and endodermal cell lineages, followed by the maturation of three-dimensional hosts (Figure S1A). As previously described,^17^ assembloid assays were carried out by co-culturing patient-derived GB and epilepsy surgery-derived (adult) brain neural progenitor (NP) spheroids with their neural and endodermal hosts, enabling a comparison of GB and NP invasion dynamics and phenotypes across two different cellular environments (Figure 1A). Green fluorescent protein (GFP)-expressing primary GB cells from the established patient-derived “workhorse” model GBM1^9^^,^^28^ readily infiltrated both neural and endodermal lineage-biased hosts. In contrast, GFP-expressing epilepsy surgery-derived NP1^28^ cells fused with the host structures but exhibited significantly reduced (≥2-fold, *p* < 0.0001) infiltration post fusion (Figure 1B). As expected, endodermal and neural GB assembloids displayed distinct morphological features (e.g., a ventricular-mimicking compartment exclusive to neural GB assembloids, Figure 1C). Endodermal GB assembloids expressed lineage-specific markers, including pancreatic and duodenal homeobox 1 (PDX1) and albumin (ALB), indicative of a mix from foregut to hepatic cell fates. In contrast, neural GB assembloids expressed SRY-box transcription factor 2 (SOX2), a neuroepithelial progenitor marker, and βIII-tubulin (TUJ1), indicative of a maturing neuronal compartment (Figure 1C).

Next, we dissociated GB-infiltrated assembloids after 72 h of culture, isolating GFP-expressing GB cells from unlabeled environmental host cells, using fluorescence-activated cell sorting (FACS). The sorted cell populations were then processed for scRNA-seq (10x Genomics), yielding 17,424 single-cell transcriptomes for analysis of gene expression dynamics in both tumor and host organoid compartments. Uniform Manifold Approximation and Projection (UMAP) visualizations (generated using the Louvain clustering with Harmony embedding)^29^ revealed that GB cells invading endodermal and neural host organoids formed neighboring subclusters, while largely remaining within the main GB cluster observed in (pre-assembly) control cells. Tumor cells were distinct from the endodermal and neural host background environments (Figures 1D, 1E, and S1B). As expected, endodermal and neural lineage environments occupied distinct spaces in the UMAP projection, exhibiting separate single-cell expression signatures (Figures 1F and S1B), whereas the UMAP visualization indicated a robust overlap between invaded and non-invaded GB assembloid environments (Figures 1E and 1F). Consistently, differential gene expression analysis revealed microenvironment-specific transcriptional responses in the host cells following GB infiltration. In endodermal environments, the infiltrated host cells upregulated genes (e.g., *LDHA*, *S100A6*, and *SPARCL1*) associated with glycolytic metabolism, calcium signaling, and extracellular matrix interaction (Figure 1G). In contrast, neural environment host cells displayed an increased expression of genes associated with immune modulation, oxidative stress, and neural lineage adaptation (e.g., *LGALS3*, *MT3*, and *C1ORF61*; Figure 1H). Notably, the expression of cyclin-dependent kinase inhibitor 2A (CDKN2A), a clinically relevant tumor suppressor frequently deleted in IDH-wildtype GB,^30^ was markedly increased in the invaded neural host environment cells.

In agreement with the expected endodermal vs. neural lineage differentiation in each GB invasion assay, UMAP of transcriptomes from the overall assembloid cell types (aligned with reference single-cell data)^31^ revealed segregation into lineage-specific clusters (e.g., neurons, neuroepithelial cells, and epithelial and endothelial populations; Figure S1B). Expression of key lineage markers assessed by RT-qPCR independently confirmed host organoid lineage identity in post-invasion GB assembloids: *SOX17* was enriched in endodermal environments, while *TUBB3* was significantly elevated in neural host conditions (both *p* < 0.001; Figure S1C). Gene Ontology (GO) enrichment analysis further supported distinct transcriptional responses to GB invasion across the two assembloid types. Endodermal cells showed enrichment for developmental and tissue remodeling programs (e.g., regionalization and mesenchymal differentiation), whereas neural cells displayed enrichment for axon development, Wnt signaling, and immune-related processes. In both environments, pre-invasion control cells were primarily enriched for cell cycle and chromosomal segregation pathways (Figures S2A and S2B).

To examine whether the invasion-associated gene expression observed in GB cells was shaped by the host environment or reflected intrinsic (cell-autonomous) properties, we compared marker gene expression across stemness, proliferation, invasion, and mesenchymal programs in GB cells isolated from neural and endodermal assembloids, alongside pre-invasion control GB cells (Figure S2C). Expression patterns were broadly similar across neural and endodermal host organoid contexts, displaying transcripts associated with invasion (e.g., MMP2, CDH2, and TGFBI), mesenchymal transition (e.g., CD44, STAT3, and TWIST1), and stemness (e.g., TRRAP), which were consistently expressed largely regardless of host organoid lineage identity, suggesting a potential cell-intrinsic and lineage-agnostic GB invasion program.

### Identification of intrinsic GB invasion-associated signature

**Figure 2:**
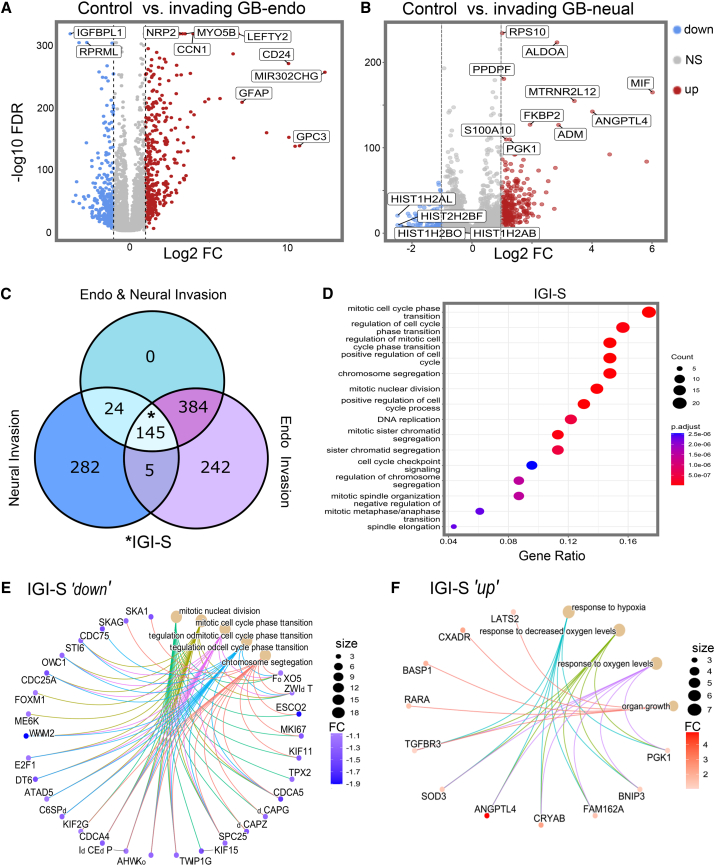
Identification of intrinsic GB IGI-S from single-cell analysis (A and B) Volcano plots showing differentially expressed genes in individual GB cells before invasion (control) and after invasion into endodermal (A) and neural (B) host environments (FDR < 0.01; log_2_ FC > 1 or < −1). (C) Venn diagram showing the overlap of differentially expressed genes across neural and endodermal invasion, defining the 145-gene IGI-S. (D) Gene Ontology biological process enrichment analysis of IGI-S genes, displaying the top 15 pathways (adjusted *p* < 0.05). (E) Cnetplot of genes in the top 5 enriched pathways among the 59 IGI-S downregulated genes. (F) Cnetplot of genes in the top 5 enriched pathways among the 86 IGI-S upregulated genes. Node size in (E) and (F) reflects gene count; color indicates fold change. See also Figure S2. IGI-S, intrinsic GB invasion-associated signature.

To identify gene expression changes associated with the invasive transition in GB assembloid cells, we compared transcriptomes of individual GB cells isolated after their infiltration into endodermal or neural host environments with pre-invasion GB control cells. Volcano plots revealed substantial transcriptional changes in both contexts (Figures 2A and 2B): in endodermal assembloids, invasion was associated with the upregulation of genes linked to extracellular interactions and morphogenesis (i.e., *NRP2*, *MYO5B*, and *CD24*), as well as developmental regulators (i.e., *LEFTY2* and *MIR302CHG*). Increased expression of glial fibrillary acidic protein (GFAP) was observed, consistent with maintenance or reactivation of astrocytic lineage identity within the tumor context. Significantly downregulated genes included those linked to growth control and differentiation (e.g., *IGFBPL1* and *RPRML*), which are relevant in cancer contexts. However, their functional relevance in GB remains largely unclear (Figure 2A). In neural host environments, GB invasion induced the expression of genes associated with metabolic adaptation and hypoxia response (e.g., *PGK1*, *ALDOA*, *ANGPTL4*, *ADM*, and *MIF*), along with stress resilience markers (e.g., CRYAB and BNIP3). A notable downregulated group included multiple histone variant genes (*HIST1H2AB*, *HIST1H2BO*, and *HIST2H2BF*), suggesting chromatin reorganization or a shift from a proliferative cell state (Figure 2B).

To determine whether a shared transcriptional program underlies GB invasion across endodermal and neural host environments, we identified the intersection of differentially expressed genes from GB cells isolated from each assembloid condition. This analysis defined a 145-gene intrinsic GB invasion-associated signature (IGI-S), comprising genes consistently regulated during invasion regardless of the endodermal and neural host organoid developmental lineage (Figure 2C). GO revealed significant overrepresentation of the processes related to cell cycle control and chromosomal segregation (Figure 2D). To resolve biological roles within the IGI-S, genes were stratified by regulation direction and visualized with cnetplots in R, using the clusterProfiler R package,^32^ which maps gene-to-pathway relationships and highlights shared biological contributions (Figure S3A). Downregulated genes within IGI-S included several mitotic-progression and spindle-assembly factors (e.g., *CDCA5*, *TPX2*, and *KIF11*; Figure 2E), Upregulated IGI-S genes were associated with hypoxia response, oxidative stress tolerance, and metabolic reprogramming (e.g., *ANGPTL4*, *PGK1*, *CRYAB*, and *SOD3*; Figure 2F), indicating a shift toward stress-adaptive states. Global visualization by t-distributed stochastic neighbor embedding (tSNE) indicated that both pre-tumor-organoid controls and the invading GB population contain a sizable G2/M-enriched fraction (∼30%; Figure S3B, defined using the G2/M gene set from Neftel et al.^33^). Upon the formation of tumor assembloids, transcriptional diversity increased (Figure S3B), with invasion-associated subclusters beginning to express G2/M markers. Together, these observations suggest that G2/M-phase cells emerge within the assembloid environment and that malignant invasion is accompanied by increased diversity in G2/M-related transcriptional states, warranting dynamic tracking of cell cycle phenotypes (explored later in this article).

To assess the potential clinical relevance of IGI-S, we performed Kaplan-Meier survival analysis using TCGA-GBM patient data,^34^ stratifying cases by expression of the broader IGI-S “up” and “down” gene sets. Expression of the 59 upregulated IGI-S genes was significantly associated with reduced overall survival (*p* < 0.001; Figure S3C), whereas no significant association was observed for the 86 downregulated genes (*p* = 0.15; Figure S3D). Together, the characterization of a 145-gene set defines IGI-S as a conserved, cell-autonomous transcriptional program underlying dynamic GB invasion into two distinct developmental lineage microenvironments.

### GB-intrinsic “HUB” subnetwork within IGI-S associates with dismal prognosis

**Figure 3:**
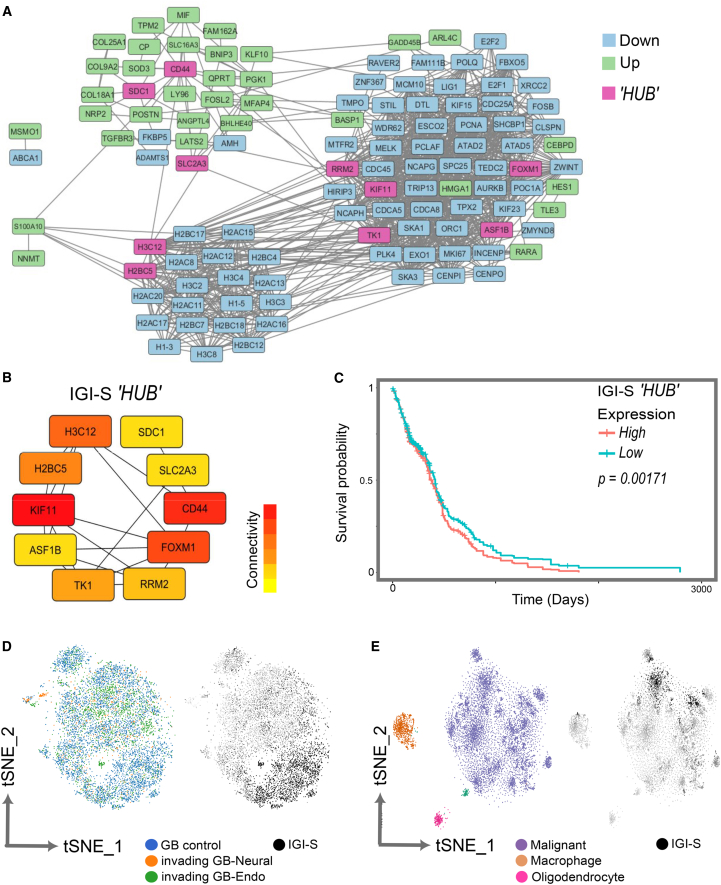
IGI-S “HUB” gene network and association with patient survival (A) STRING PPI network of IGI-S genes. Nodes are colored by expression direction: upregulated (green), downregulated (blue), and “HUB” genes (pink); edges represent predicted interactions. (B) Ten IGI-S “HUB” genes identified from the 145-gene IGI-S set using cytoHubba,^36^ visualized by connectivity (color scale). (C) Kaplan-Meier overall survival analysis of TCGA-GBM patients stratified by high vs. low IGI-S “HUB” gene signature expression (*p* = 0.00171). (D) tSNE plot of GB assembloid (tumor) cells colored by condition (left; control, neural invasion, endodermal invasion) and IGI-S hub gene expression (right; black, IGI-S-expressing cells). (E) tSNE plot of GB cells from Neftel et al.^33^ annotated by cell type (left) and IGI-S hub gene expression (right; black, IGI-S-expressing cells). See also Figure S3. IGI-S, intrinsic GB invasion-associated signature; PPI, protein-protein interaction; tSNE, t-distributed stochastic neighbor embedding.

To prioritize functionally central components within the IGI-S invasion program, we constructed a protein-protein interaction (PPI) network across all 145 IGI-S genes using Search Tool for the Retrieval of Interacting Genes/Proteins (STRING)^35^ (Figure 3A), followed by network topology analysis with cytoHubba.^36^ This approach identified a ten-gene IGI-S “HUB” module comprising both upregulated and downregulated genes with high PPI connectivity (Figure 3B). The identified factors included known regulators of cell cycle and invasion (e.g., FOXM1, CD44, KIF11, and RRM2) as well as genes involved in metabolism and transport (*SLC2A3*, *ASPM*, and *SDC1*), suggesting the integration of proliferative, migratory, and metabolic features within the GB invasion program. Notably, high expression of the ten-gene IGI-S “HUB” signature was also significantly associated with poorer survival (*p* = 0.00171; Figure 3C), supporting a prognostic IGI-S potential.

To evaluate IGI-S heterogeneity in a single-cell context, we first mapped IGI-S gene expression across our GB scRNA-seq datasets. tSNE projections demonstrated that IGI-S expression was confined to a subset of GB cells infiltrating both endodermal and neural assembloids (Figure 3D), consistent with inter-tumoral heterogeneity and the influence of additional environmental cues on other invading populations. Finally, we mapped IGI-S expression to previously published single-cell patient tumor reference data from Neftel and colleagues.^33^ Cell type classification confirmed that IGI-S expression was restricted to malignant cells, with no enrichment in tumor-associated macrophages or oligodendrocytes (Figure 3E). Within the malignant compartment, IGI-S expression was confined to a small subpopulation (∼20% of total tumor cells), suggesting that IGI-S marks a distinct, potentially aggressive sub-lineage within a broader patient tumor single-cell pool.

Overall, these findings support the notion that IGI-S may represent a tumor-intrinsic, invasion-linked transcriptional program enriched in a malignant subpopulation. Identification of such IGI-S at single-cell resolution within our lineage-modulated GB assembloid system highlights the utility of this 3D tumor model platform for mimicking patient-like cellular plasticity during the early and dynamic stages of GB invasion.

### GB assembloids enable real-time visualization of cell cycle phases

**Figure 4:**
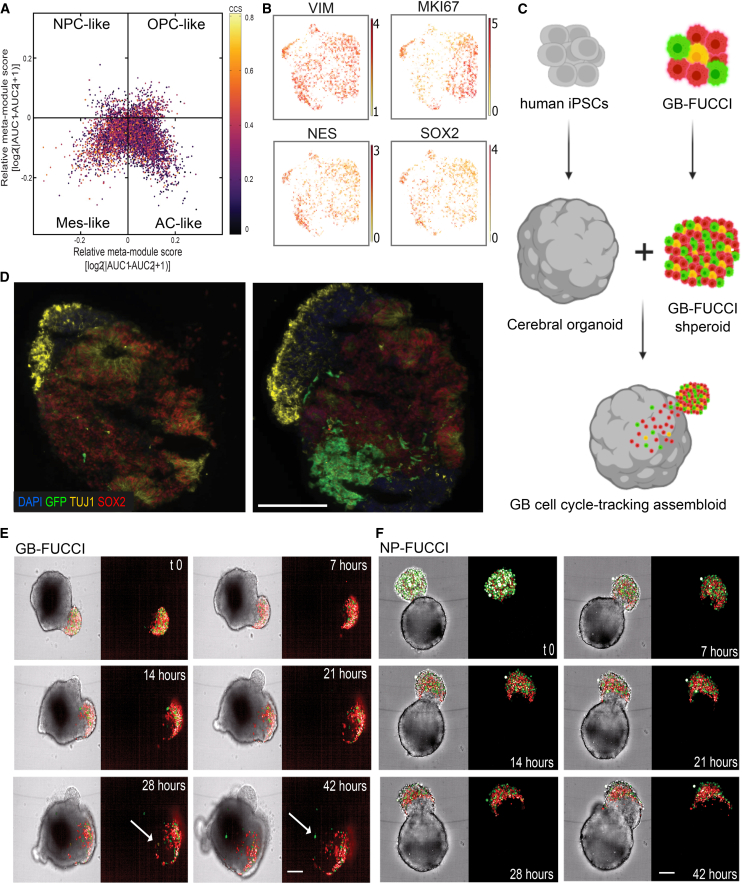
GB-FUCCI assembloids recapitulate tumor invasion phenotypes (A) Two-dimensional projection of single-cell transcriptomes from GB-FUCCI cells (derived from the GBM1 line),^28^^,^^37^ classified into developmental-like GB cellular plasticity states—neural progenitor (NPC), oligodendrocyte progenitor (OPC), mesenchymal (Mes), and astrocytic (AC).^33^ Color bar denotes the cell cycle score (CCS), a proxy for proliferation activity. (B) Two-dimensional (UMAP) projection of cells colored by expression of the indicated marker gene, with the color scale indicating log-normalized expression levels. (C) Schematic for the generation of GB-FUCCI assembloids by co-culture of tumor spheroids with human COs. (D) Immunohistochemical staining of COs alone or co-cultured with infiltrating GFP-expressing GB cells, showing expression of the indicated proteins. Scale bars: 200 μm. (E) Time-lapse confocal microscopy showing progressive infiltration of GB-FUCCI cells into a CO. Each frame is displayed as a brightfield/fluorescence overlay (left) and fluorescence-only image (right). Final frames highlight an infiltrative cell (arrow) migrating in G2/M phase. Scale bars: 200 μm. (F) Time-lapse microscopy showing minimal infiltration of NP-FUCCI non-cancerous control cells (derived from NP1 line)^28^ into a CO. Scale bars: 200 μm. See also Figure S4.

Our single-cell transcriptomic analysis of lineage-modulated assembloids revealed both intrinsic and environment-responsive regulation of cell cycle pathways during GB invasion. In line with these observations—and with the increasingly questioned role of GB proliferation in invasion^27^—we developed DyPheT, an automated tracking tool for longitudinal mapping of cell cycle progression at single-cell resolution during infiltration by individual tumor cells into COs. To this end, using scRNA-seq, we first characterized the baseline transcriptional states of our GB-FUCCI workhorse model GBM1,^24^^,^^27^ which we employed throughout the DyPheT development approach. Two-dimensional projection of the single-cell transcriptomes revealed heterogeneous plasticity and proliferation states of the cell model, with GB-FUCCI cells enriched in mesenchymal-like and astrocytic-like profiles, and comparatively fewer cells in NP-like and oligodendrocyte progenitor-like phenotypes^33^ (Figure 4A). Expression of selected molecular markers (e.g., VIM, SOX2, CD44, and GFAP) further confirmed this pattern (Figures 4B and S4A).

To investigate how cell cycle progression relates to the malignant infiltration behavior of individual GB cells, we established a CO-based assembloid model suitable for live-cell tracking (Figure 4C). Early-stage COs, with or without the tumor compartment, displayed reproducible structural organization, including central SOX2-positive neuroepithelial zones and peripheral TUJ1-positive neuronal maturation compartments, and served as the host environment for invasion assays (Figure 4D). Time-lapse confocal microscopy revealed progressive infiltration of GB-FUCCI cells into the CO compartment. A subset of highly infiltrative GB-FUCCI cells was observed in the proliferative (green-appearing) G2/M phase of the cell cycle, supporting a potential functional link between cell cycle progression and GB invasion behavior (Figure 4E). In contrast, NP-FUCCI control cells (derived from the adult brain progenitor line NP1)^28^ exhibited minimal invasion (Figure 4F). Consistent with their non-malignant phenotype, NP-FUCCI cells showed significantly reduced migration compared with their GB-FUCCI counterparts derived from three independent patient-derived models (GBM1, GBM4, and GBM20)^28^ at 24 and 48 h following tumor spheroid-CO interaction (Figure S4B).

### Correcting 3D movement and rotation enables automated quantification of cell cycle-dependent infiltration

**Figure 5:**
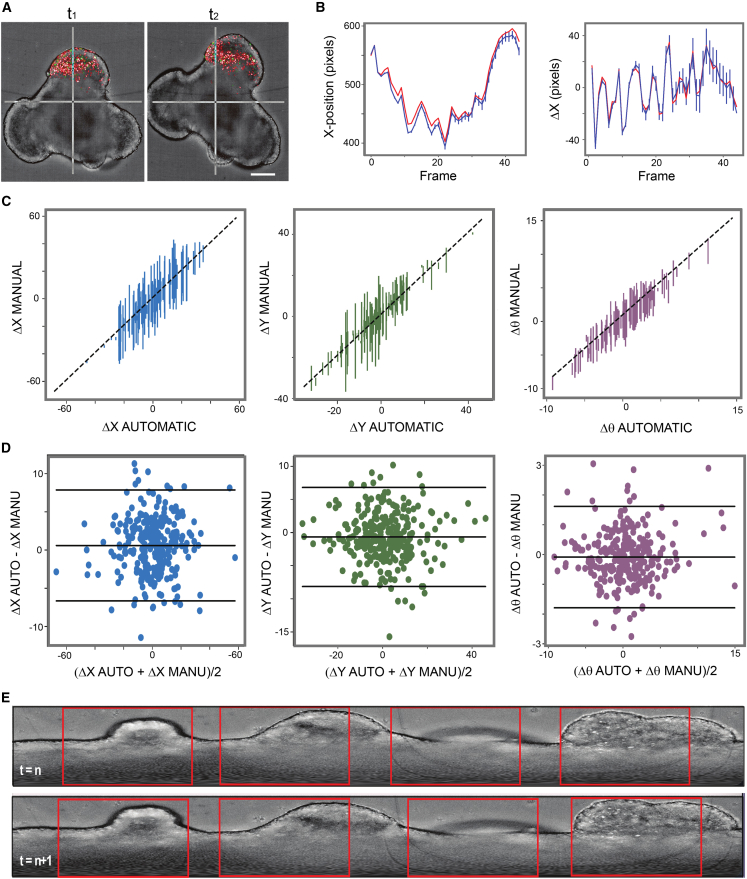
Developing automated assembloid tracking in DyPheT (A) Time-lapse frames of GB assembloids. t_1_: 32 h of co-culture between a GB-FUCCI (fluorescent ubiquitination-based cell cycle indicator) spheroid and a CO. t_2_: 48 h of co-culture, showing rotational movement and morphological changes in the assembloid, necessitating frame-to-frame correction. Scale bars: 200 μm. (B) Each frame was aligned to the previous one through translation and rotation, either manually by three independent validators (average shown as blue line with standard error bars) or automatically by using DyPheT (red line). Positional correction involved adjustment of *x* position (shown here), *y* position, and rotation angle (*θ*) for one selected video, with delta parameter comparisons (*ΔY* and *Δθ*) provided in Figure S5; *ΔX* is depicted in the right. (C) Correlation between the average manual and automated (DyPheT-derived) adjustments for *ΔY*, *ΔX*, and *Δθ* across five independent GB assembloid invasion assay videos. The dashed line represents the line of best fit, indicating strong agreement between manual validation and automated tracking. (D) Bland-Altman plots showing the difference between manual and DyPheT-derived values for *ΔX*, *ΔY*, and *Δθ* across five independent videos. Solid black lines represent the mean difference (center) ±2 standard deviations (limits of agreement). (E) Representative consecutive time-lapse frames (t = n, t = n+1) showing polar-unwrapped views of the GB assembloid surface, where the height represents the radius from the center of assembloid and the horizontal represents *θ*. Red boxes mark defined segments used to compare changes between frames. See also Figure S5.

Quantitative analysis of single-cell dynamics as a function of cell cycle phase from time-lapse imaging of GB assembloids posed specific challenges due to the dynamic nature of the free-floating, low-adherence culture conditions. Inevitably, the two interacting spheroidal structures (GB and COs) undergo continuous translational and rotational motion before and after fusion, as well as volumetric expansion as GB assembloids over the course of the assay (Figure 5A). These global assembloid movements obscure the independent trajectories of infiltrating tumor cells, necessitating computational correction to enable accurate single-cell tracking over time.

To address this, we developed an automated framework that integrates mathematical modeling into DyPheT to correct for translational (*x* and *y*) and rotational (*θ*) assembloid displacements between timepoints (Methods S1), enabling spatially and temporally resolved extraction of single-cell trajectories and hue-based, color-coded cell cycle states. Video frames (acquired every 60 min) were aligned based on frame-to-frame shifts, with DyPheT estimating the translational and rotational parameters required to register each frame to its predecessor. Correction accuracy was benchmarked against manual alignment performed independently by three validators, with comparisons made between automated and averaged manual adjustments (Figure 5B). The results indicated strong concordance between DyPheT-derived tracking curves and manually derived *x*, *y*, and *θ* corrections (Figures 5B and S5). Minor deviations were observed, including a gradual drift in the automated *y*-alignment relative to manual tracking. However, analysis of frame-to-frame displacements (*ΔX*, *ΔY*, and *Δθ*) over time showed that automated corrections closely matched manual adjustments, typically deviating by only a few pixels or, in the case of rotation, by less than 1°.

Consistently, quantitative correlation analysis of frame-to-frame displacements across five independent time-lapse videos demonstrated high agreement, with Pearson’s correlation coefficients of 0.99 (*ΔX*), 0.86 (*ΔY*), and 1.00 (*Δθ*) (Figure 5C). Bland-Altman analysis further supported agreement between the manual and DyPheT correction methods, treating manual alignment as a reference standard. The majority of data points fell within ±2 standard deviations of the mean difference, which was close to zero in all cases, indicating the absence of systematic bias in the automated relative to manual alignment (Figure 5D). Moreover, DyPheT applied polar unwrapping that linearizes the curved assembloid surface, allowing direct, segment-wise comparison of local morphological features between consecutive frames (Figure 5E). These results establish DyPheT as a robust and unbiased approach for correcting rotational and translational motion in dynamic 3D time-lapse imaging of GB assembloids, enabling the extraction of cell trajectories and phenotypic transitions at single-cell resolution.

### DyPheT tracks “go or grow” and “go and grow” GB cell fate decisions

**Figure 6:**
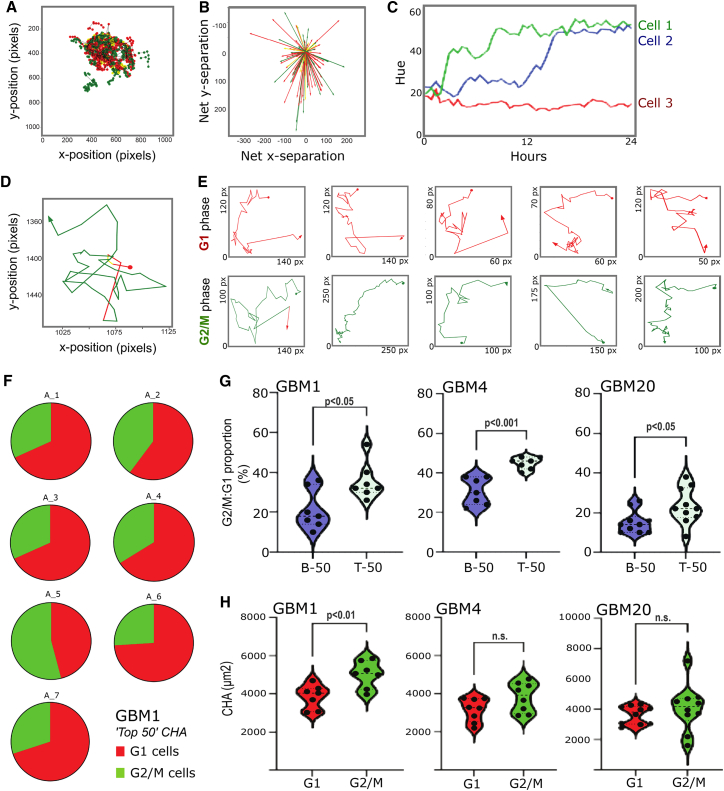
DyPheT distinguishes between “go or grow” and “go and grow” tumor cell fate decisions in GB assembloids (A) DyPheT-derived trajectory map of individual tumor cells invading the GB assembloids, displaying cell cycle states, color coded according to the FUCCI system64; G1, red; S, yellow; and G2/M, green. (B) Directional migration vectors for individual GB-FUCCI cells within assembloids, color coded by cell cycle state (G1, red; S, yellow; G2/M, green), revealing cell motility linked to cell cycle phases. (C) Example of DyPheT-based hue tracking in three individual GB-FUCCI cells. Each begins near hue 20 (red/yellow; G1). Cell 1 (green) and cell 2 (blue) show hue increases to ∼50–60, consistent with S/G2/M entry. Cell 3 (red) maintains a stable hue value, indicating prolonged G1 phase. (D) Two-dimensional DyPheT plot of a single GB-FUCCI cell trajectory, integrating spatial positioning with dynamic transitions between G1 (red) and G2/M (green) states. (E) Representative examples of migratory trajectories of GB-FUCCI cells, either predominantly in G1 phase (top) or in G2/M phase (bottom), revealing differential cellular motility. (F) Pie charts showing the relative proportions of G2/M (green) and G1 (red) phase cells within the top 50 cells ranked by CHA score in seven independent GBM1 assembloids (A1–A7). Top 50 CHAs were derived from an average of 225 G1 and 81 G2/M cells per assembloid. (G) Violin plots comparing G2/M-to-G1 cell ratios in bottom 50 (B-50) versus top 50 (T-50) CHA-ranked populations across three GB models (GBM1, GBM4, and GBM20), with individual cell data shown as dots. (H) Violin plots comparing migration distances between G1 and G2/M phase cells in GB assembloids derived from GBM1 (*n* = 7), GBM4 (*n* = 8), and GBM20 (*n* = 10) models. Statistical comparisons were performed using unpaired two-tailed Student’s *t* tests. See also Figure S6.

Following automated orientation correction of GB assembloids using high-throughput confocal time-lapse imaging data, we applied DyPheT to track single tumor cell movements within the 3D structures. In contrast to the less coordinated, multidirectional migration observed in adherent cultures, GB cells exhibited more directed trajectories in assembloids, accompanied by reduced angular movement heterogeneity (Figure S6A). Using FUCCI-labelled cells, DyPheT generated infiltration trajectory maps (Figure 6A) and directional vector maps (Figure 6B), both enriched with color-coded cell cycle phase information. This enabled dynamic interpretation of invasive GB behavior with regards to “go or grow” versus “go and grow” cell fate decisions. Furthermore, cell cycle transitions can be longitudinally quantified from hue values extracted from FUCCI-GB cells, where hue encodes fluorescence signal ratios corresponding to G1, S, and G2/M phases (Figure 6C). Time-lapse imaging was performed at 60 min intervals across multiple confocal planes (per-well scan duration <15 min) to balance temporal resolution and data volume while keeping phototoxic effects negligible.^38^ DyPheT enables visualization of these dynamic cell cycle changes and generates two-dimensional trajectory maps^39^ that track phase transitions along individual migratory paths, providing an integrated spatiotemporal representation of tumor cell behavior (Figures 6D and 6E).

We analyzed both the distance traveled by individual cells and their convex hull area (CHA) as a surrogate measure of cellular migration,^40^ fueling assembloid-invasion capacity of the GB cells. In this context, the CHA reflects the two-dimensional area spanned by the entire trajectory of a FUCCI-labelled cell that captures the overall migratory behavior more comprehensively than linear distance alone. To identify highly migratory subpopulations, we ranked all cells by CHA within each GB assembloid and compared the proportion (%) of G2/M versus G1 phase cells among the top 50 and bottom 50 ranked cells. Pie charts were created to illustrate the relative composition of G2/M and G1 cells among the top 50 CHA-ranked cells in GBM1 assembloids, revealing that G1-phase cells predominated in 6 out of 7 assembloids (Figure 6F). However, violin plots showed a bimodal distribution of mean CHA values across the three different FUCCI-labelled GB cell models. Notably, these data revealed a significantly higher G2/M-to-G1 ratio among the top 50 CHA-ranked cells compared with the bottom 50, suggesting the existence of a subpopulation of highly infiltrative GB cells in the G2/M phase of the cell cycle (Figure 6G).

Comparison of both mean distance traveled and mean CHA values between G1-and G2/M-phase cells across three GB-FUCCI models demonstrated that G2/M cells are at least as migratory and invasive as G1 cells (Figures 6H and S6B). Together, these results indicate that GB cells engage in a mixture of “go and grow” and “go or grow” behaviors during assembloid invasion. This is consistent with a ∼25% MKI67-positive tumor cell fraction that was observed across three independent recurrent GB specimens obtained specifically from the peripheral tumor regions (Figures S6C and S6H). Furthermore, these findings align with the conservation of a G2/M-associated signature^33^ and are evident within heatmap-based visualization of Darmanis et al.’s dataset (Figure S6E),^41^ in which single-cell expression profiles from peripheral tumor regions show enrichment of proliferative states relative to the tumor core, as well as with observations from murine GB invasion models.^27^ In addition, our DyPheT analysis identifies a distinct subpopulation of highly migratory G2/M-phase cells, revealing a proliferative cell state associated with enhanced invasive potential during the early stages of 3D tumor infiltration *ex vivo*.

### DyPheT identifies proliferation and migration changes in GB assembloids upon chemical cell cycle modulation

**Figure 7:**
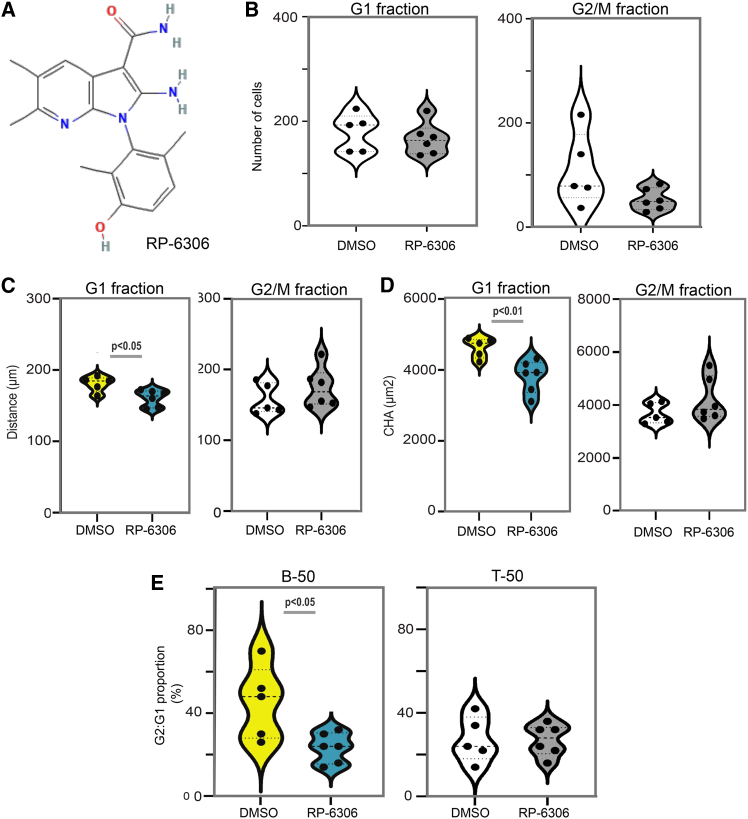
DyPheT quantifies cell cycle-specific migration changes in GB-FUCCI assembloids treated with RP-6306 (A) Chemical structure of RP-6306. (B) RP-6306 treatment (50 nM) did not significantly alter the total number of cells in the G1 or G2 phase compared with the DMSO control (0.0005%). (C) Migration distance of G1 phase cells was significantly reduced upon RP-6306 treatment, while G2 phase cell migration remained unchanged relative to DMSO. (D) CHA was significantly decreased in G1 phase cells following RP-6306 exposure, with no effect observed in G2 cells. (E) Ranking the top 50 (T-50) and bottom 50 (B-50) cells by CHA across DMSO- and RP-6306-treated assembloids revealed a significant reduction in the G2:G1 cell ratio within the B-50 population upon RP-6306 treatment, but not in the T-50 group. Statistical comparisons were performed using unpaired, two-tailed Student’s *t* tests. Dots represent individual GB-FUCCI assembloids. Violin plot horizontal lines indicate the median and interquartile ranges. Sample sizes: DMSO, *n* = 5; RP-6306, *n* = 6. Gray-scale violin plots denote non-significant comparisons; colored plots highlight statistically significant differences (*p* values as shown). See also Figure S7.

To assess the potential of real-time phenotype tracking in GB assembloids for compound screening, we conducted a proof-of-concept experiment using RP-6306 (Figure 7A), a selective inhibitor of protein kinase membrane associated tyrosine/threonine 1 (PKMYT1).^42^ PKMYT1 negatively regulates CDK1 activity through phosphorylation,^43^ thereby maintaining the G2/M checkpoint. Inhibition by RP-6306 forces premature mitotic entry, leading to chromosomal instability and context-dependent cell death. For example, RP-6306 has demonstrated activity in CCNE1-amplified cancers^44^ and has been evaluated in GB models in combination with temozolomide.^45^

We employed the GB-FUCCI model (using the GBM1 line) to assess whether DyPheT could detect RP-6306-induced changes in cell cycle phase distribution and tumor cell migration, as a test for dynamic, phenotype-based compound evaluation. Five GB assembloids were treated with DMSO, and six were treated with RP-6306. At the 65 h endpoint, time-lapse data were analyzed using DyPheT to quantify total infiltrating GB cells and assess their migration using CHA. No statistically significant differences were observed between the control and RP-6306-treated groups in terms of the average cell number or CHA-derived migration features per assembloid (Figures S7A and S7B). To further assess whether RP-6306 alters tumor cell migration in a cell cycle-dependent manner, we performed a stratified analysis of G1- and G2/M-phase fractions across GB assembloids. The total number of tracked GB-FUCCI cells did not differ significantly between G1 and G2/M cells within either treatment group (Figure 7B). However, in G1 cells, RP-6306 treatment significantly reduced the average migration distance from 180.8 μm (DMSO control) to 160.6 μm (*p* < 0.05), while no significant change was observed in G2/M cells (Figure 7C). Similarly, CHA-defined area coverage in G1 cells decreased from 4,634 μm^2^ to 3,817 μm^2^ under RP-6306 treatment (*p* < 0.01), with no corresponding difference in G2/M cells (Figure 7D). In addition, the G2/M-to-G1 ratio among the bottom 50 CHA-ranked cells was significantly reduced following RP-6306 treatment, dropping below 40% (*p* < 0.05; Figure 7E), while no change was observed in the top 50 CHA-ranked cells across control and treated assembloids.

These results suggest that RP-6306 reduces GB assembloid invasion by lowering the migration behavior of G1-phase cells and altering the cell cycle phase composition of the low-migratory tumor cell population, while leaving the highly migratory G2/M-enriched fraction unaffected, suggesting that this subpopulation may be more resistant to cell cycle checkpoint modulation. The ability of DyPheT to resolve parameters supports its utility for cell cycle-resolved and phenotype-based assessment of compound effects and potential screening applications.

## Discussion

A broad range of preclinical GB models based on rodent, zebrafish, and *Drosophila melanogaster* have provided important insights into GB invasion, with their strengths and limitations discussed in recent reviews.^46^^,^^47^^,^^48^ Within this landscape, organoid-based systems have become tractable tools for real-time as well as longitudinal (endpoint) analysis of GB invasion in a human cellular context, for example using iPSC-derived neural or COs co-cultured with patient-derived tumor cells.^17^^,^^18^^,^^19^^,^^20^^,^^22^^,^^49^^,^^50^ These assembloid models replicate key features of early tumor–host interactions and are widely used to study GB infiltration in brain-like environments.^50^ Recent advances in patient-derived tumor organoid systems further demonstrate that organoid-based platforms can faithfully preserve human brain tumor ecosystems and can generate patient-specific treatment response predictions within clinically actionable timelines (over several days rather than several weeks), which is difficult to achieve with many other model systems.^22^ Moreover, single-cell transcriptomic studies have further advanced our understanding of GB biology in organoid contexts; for example, Krieger et al. demonstrated that transcriptional changes associated with invasion are consistent across patient-derived samples,^19^ suggesting a shared gene expression program enabling tumor cell infiltration into healthy tissue.

Recently, a body of literature highlighted the importance of neuronal niches^3^ and other brain tumor microenvironments (e.g., extracellular matrix and vasculature), fueling GB invasion.^51^ However, most current GB organoid and assembloid models are limited to neural lineage environments, making it difficult to distinguish cell intrinsic invasion mechanisms from those shaped by microenvironmental cues. While the neural lineage is the native context of GB, incorporating additional lineage environments such as foregut or hepatic organoid tissues is a biomedical imperative for testing whether GB invasion is maintained outside the neural niche.

To address this, we developed discrete assembloid models in which the invading GB cells were exposed to transcriptionally and developmentally distinct host tissues: early stage neural and endodermal. Neural host models contained neuroepithelial cells and maturing neuronal cell compartments, whereas endodermal host models displayed lineage-biasing toward foregut/hepatic identity. Despite these differences, GB cells, but not NP cells, consistently infiltrated both environments, providing proof-of-concept for a robust cell autonomous invasion capacity that is independent of host organoid lineage identity.

Single-cell transcriptomic profiling of GB-endodermal and GB-neural assembloids revealed a cell-intrinsic invasion program, termed IGI-S. This 145-gene signature was consistently expressed in GB cells across both host organoid lineages, indicating a stable invasive cellular phenotype independent of the neural tissue context. A ten-gene “HUB” subnetwork within IGI-S was enriched in malignant subpopulations in patient-derived single-cell transcriptomic data^33^ and correlated with poor prognosis, suggesting translational potential in predicting aggressive and infiltrative tumor behavior. IGI-S or related programs may contribute to the extremely rare occurrence of extracranial GB metastases, which have been described in individual case reports (including GB liver metastasis).^23^^,^^24^^,^^25^ Circulating GB cells have been detected in peripheral blood,^52^^,^^53^ suggesting that non-neural tissues could more frequently serve as GB metastatic sites in patients whose intracranial disease is more effectively controlled by next-generation therapies. The patient tumor-organoid assembloid system, in which GB cells encounter lineage-defined model tissues, allows us to test the intrinsic capacity of GB cells to infiltrate independent of their natural niche and to address fundamental questions regarding the rarity of GB metastasis and the mechanisms that may require inhibition for therapeutic targeting. Accordingly, as anti-tumor treatments improve and patient survival extends, distinguishing cell-autonomous invasion mechanisms from environment-specific adaptations may become increasingly important in a clinical context.

While IGI-S was consistently maintained across both neural and endodermal lineage contexts, GB cells also exhibited the expected microenvironment-specific gene regulation. In neural hosts, GB cells showed upregulation of immune-modulatory and neurovascular-associated genes (e.g., *MIF*, *ANGPTL4*, and *ADM*), likely driven by paracrine (stress-response/hypoxic) cues. In contrast, endodermal environments induced the expression of epithelial and hepatic lineage-associated genes (e.g., *LEFTY2*, *CD24*, *GPC3*, and *MIR302CHG*), likely reflecting a transcriptional shift in response to developmental signals. This supports the notion of lineage plasticity in GB cells extending beyond canonical neural phenotypes (e.g., astrocytic and oligodendrocytic) when exposed to non-neural tissue contexts.

In parallel, host organoid cells exhibited lineage-specific changes following GB invasion. Endodermal hosts upregulated metabolic and extracellular matrix-associated genes (e.g., *LDHA*, *SLC16A3*, and *SPARCL1*), while neural hosts activated stress-response genes (e.g., *LGALS3*, *MT3*, and *CRYAB*), alongside cyclin-dependent kinase inhibitor 2A (CDKN2A). CDKN2A is a negative regulator of the G1-S phase transition and is frequently inactivated in GB through homozygous deletion.^30^ Its upregulation in the neural assembloid environment following GB invasion may reflect a checkpoint adaptation in maturing neuronal cells, pointing to a potential link between tumor cell infiltration and host cell cycle control. However, the most significant enrichment of cell cycle-related genes was observed within the IGI-S program itself, highlighting the underexplored relationship between invasive tumor phenotypes and cell cycle progression. This gap in linking tumor cell cycle oscillation and invasion at single-cell resolution, in real time, motivated the development of the DyPheT tool.

While other studies have described long-term imaging of immobilized COs using microplate inserts,^54^ our dynamic phenotype tracker DyPheT enables automated tracking of individual cell motility in conjunction with reporter-based cell cycle state analysis in free-floating GB assembloids. DyPheT builds on prior observations of self-organizing GB invasion in murine iPSC-derived brain organoids^17^ and was here successfully adapted to a method utilizing (early-stage) human iPSC-derived COs.^55^ The migration patterns observed with DyPheT resembled the “flock”-like collective movement of GB cells described by Comba et al., potentially capturing margin zone behaviors that remain largely inaccessible to clinical imaging, yet are evident as “oncostreams” in resected tumors.^56^

DyPheT corrects the translational and rotational drift in time-lapse datasets and enables precise tracking of FUCCI-labelled GB cells at single-cell resolution. In three patient-derived GB-FUCCI models, DyPheT consistently revealed heterogeneous invasion behaviors. G1-phase cells displayed both moderate and high motility throughout experiments. Remarkably, a previously unrecognized population of highly invasive G2/M-phase cells was identified and found to be consistently enriched among the most motile and directionally migrating cells across two primary and one recurrent (patient-derived) GB cell modeling contexts, despite substantial differences in parental tumor treatment history, genotype, and molecular subtype. These findings align with intravital imaging data from Ratliff et al.,^27^ which demonstrated the co-occurrence of invasion and proliferation in GB in mice. Our results extend this concept into an expandable human stem- and patient-derived cellular assay, supporting a “go and grow” model in contrast to an exclusively “go or grow”-based tumor invasion paradigm.^26^ The presence of motile G2/M-phase cells raises the possibility that polyploid and checkpoint-insensitive subpopulations may contribute to tumor repopulation and GB recurrence, particularly arising from and within the resection margin after surgical tumor debulking. This finding also underscores the timely need for analytical tools that can capture real-time dynamics in complex organoid and/or assembloid environments. While recent work has focused on mapping the cellular architecture and topography of organoids,^57^ tools specifically designed to resolve tumor cell invasion phenotypes in real time are lacking. In this context, DyPheT can offer a broadly applicable framework for studying dynamic single-cell behavior in complex tissues, with a potential to inform machine learning^58^ and/or computational models of brain tumor invasion.^59^

In proof-of-concept drug testing, RP-6306, a selective PKMYT1 inhibitor with known anti-cancer properties,^42^^,^^44^^,^^45^ reduced motility and CHA of G1-phase GB cells but had no significant effect on the highly migratory G2/M-phase fraction. This suggests potential evasion of checkpoint regulation during GB invasion and highlights a dynamic phenotype not captured by static transcriptomic or histology profiling. It remains an open question as to whether IGI-S or comparable signatures driving invasion in an environment-agnostic fashion are functionally linked to the highly migratory G2/M cellular subpopulation. Resolving these distinct invasive behaviors may ultimately require approaches that integrate dynamic phenotyping with endpoint molecular profiling. One potential direction may be to couple live imaging-based single-cell trajectories with longitudinal spatial transcriptomic or proteomic mapping of the same cells at their final assembloid positions. Although technically demanding, such integrated workflows may allow invasion dynamics to be linked directly to spatially resolved gene expression programs further enhancing brain tumor assembloid models. Although these methods are not yet standard in assessing GB *ex vivo*, the foundation laid here provides a pathway for future integration of real-time and endpoint single-cell phenotyping within the same GB assembloid.

In conclusion, the GB assembloid approaches described here are compatible with single-cell profiling, high-content imaging, and longitudinal as well as endpoint analyses with or without experimental perturbations. Their combined application offers a phenotypically rich and mechanistically informative framework to study GB invasion in distinct host environments, supporting the identification of context-specific, cell-autonomous, and cell cycle-dependent molecular vulnerabilities.

### Limitations of the study

Several limitations remain. The models presented here do not fully reflect tumor invasion within a physiological human tissue context, as they lack vascular, immune, and other systemic components that shape the transcriptional microenvironment of GB *in situ*. While the assembloid system allows controlled interrogation of tumor-host interactions across defined developmental lineages, future refinements could include reactive astrocytes, microglia, and immune cells to better reflect post-resection microenvironments, including astrogliosis and/or fibrotic tissue changes induced by radiotherapy.^60^ However, even in the absence of immune cells, GB cells in co-culture have been shown to adopt immune- and stem-like transcriptional states,^61^ indicating that patient-derived tumor cells can retain relevant plasticity in simplified *ex vivo* systems.

In addition, employing more developmentally advanced neural and endodermal organoids may enhance the applicability of assembloid models for studying gut-brain axis biology,^62^ in particular when combined with microbiota-mimicking co-culture systems.^63^ Expanding assembloids to include additional tissue types (e.g., liver, kidney, lung, and bone tissues) could also facilitate the investigation of metastatic routes and tissue-specific colonization patterns of non-CNS tumors such as breast cancer or melanoma, including their propensity to invade the brain compared to other organs. In this study, our choice of model system was driven by the need for real-time observation of tumor cell invasion behavior and precise iPSC-based lineage specification of “host” organoid *in vitro* models, together with the ability to link these dynamic phenotypes to scRNA-seq endpoint readouts. Orthotopic xenograft models offer complementary advantages, including a vascularized host environment, a multicellular tissue context, and mammalian brain anatomy, all of which will be important for extending our findings *in vivo*. Future work may include orthotopic xenograft experiments to validate IGI-S as identified by scRNA-seq analysis and to assess the robustness of the highly migratory G2/M cell subpopulation identified by DyPheT.

## Resource availability

### Lead contact

Further information and requests for resources and reagents should be directed to and will be fulfilled by the lead contact, Heiko Wurdak (h.wurdak@leeds.ac.uk).

### Materials availability

All unique resources and reagents generated from this study are available from the lead contact upon request, subject to completed materials transfer agreement.

### Data and code availability

• The mRNA-seq data (FASTQ files) have been deposited in the NCBI Gene Expression Omnibus (GEO), with accession number GEO: GSE298718, and are publicly available. The link is provided in the key resources table. Data reported in this paper will be shared by the lead contact upon request.
• The DyPheT tool and code for automated assembloid tracking are available via GitHub. The link is provided in the key resources table.
• Additional information required to reanalyze the data reported in this paper are available from the lead contact upon request.

## Acknowledgments

We thank Hazel Fernando for assistance with GEO data deposition. We thank Isaac Critchley for providing additional independent manual verification for the DyPheT tool. We thank the Leeds Neuropathology Research Tissue Bank and Steven Pollock for assisting with anonymized demographic sample information. C.Y.A.-F. was supported by the 10.13039/501100000297Royal College of Surgeons of England (The George Drexler Foundation) and the Leeds Hospital Charity doctoral fellowship. B.K.I., R.K.M., and H.W. were supported by a Cheney research award (10.13039/501100000777University of Leeds). H.W. and R.K.M. were also supported by 10.13039/501100000266EPSRC IRC in Targeted Delivery for Hard-to-Treat Cancers funds, and R.K.M. acknowledges funding from Yorkshire’s 10.13039/501100002203Brain Tumour Charity, Candlelighters and 10.13039/100013790Brain Research UK.

## Author contributions

Conceptualization, C.Y.A.-F., B.K.I., A.I., D.M.A.G., K.C., R.K.M., and H.W.; methodology, C.Y.A.-F., B.K.I., A.I., S.S., E.S., S.B., A.B., I.M.H., D.M.A.G., K.C., R.K.M., and H.W.; validation, C.Y.A.-F., B.K.I., A.I., S.S., E.S., S.B., A.B., I.M.H., D.M.A.G., K.C., R.K.M., and H.W.; formal analysis, C.Y.A.-F., B.K.I., A.I., S.S., E.S., S.B., A.B., I.M.H., D.M.A.G., K.C., R.K.M., and H.W.; investigation, C.Y.A.-F., B.K.I., A.I., S.S., E.S., H.E.B., S.B., A.B., I.M.H., D.M.A.G., K.C., R.K.M., and H.W.; resources, I.M.H., K.C., R.K.M., and H.W.; data curation, C.Y.A.-F., B.K.I., A.I., K.C., R.K.M., and H.W.; writing – original draft, C.Y.A.-F., A.I., and H.W.; writing – review & editing, C.Y.A.-F., B.K.I., A.I., S.S., E.S., H.E.B., S.B., A.B., I.M.H., D.M.A.G., K.C., R.K.M., and H.W.; visualization, C.Y.A.-F., B.K.I., A.I., S.S., E.S., and H.W.; supervision, I.M.H., D.M.A.G., K.C., R.K.M., and H.W.; project administration, K.C., R.K.M., and H.W.; funding acquisition, R.K.M. and H.W.

## Declaration of interests

The authors declare no competing interests.

## STAR★Methods

### Key resources table

**Table:** 

REAGENT or RESOURCE	SOURCE	IDENTIFIER
**Antibodies**		

SOX2	Cell Signaling	Cat# 3579S
TUJ1	BioLegend	Cat# 801202
GFP	Abcam	Cat# ab13970
MKI67	Abcam	Cat# ab15580
Anti-chicken Alexa 488	Invitrogen	Cat# A11039
Anti-rabbit Cy3	Jackson ImmunoResearch	Cat# 711-165-152
Anti-mouse Alexa 647	Invitrogen	Cat# A21235

**Recombinant DNA**		

CMV-TurboGFP-Puro Lentiviral Vector	Sigma-Aldrich	Cat# SHC003V
FUCCI (LTV-0052-1) Lentiviral Vector	LipExoGen	Cat# LTV-0052-1

**Chemicals, peptides, and recombinant proteins**		

Poly-L-ornithine	Sigma	Cat# P3655
Laminin	Invitrogen	Cat# 23017-015
Vitronectin	Gibco	Cat# A14700
Neurobasal medium	Gibco	Cat# 21103-049
B-27 supplement	Invitrogen	Cat# 17504-044
*N*-2 supplement	Invitrogen	Cat# 17502-048
Recombinant human bFGF	Gibco	Cat# PHG0026
Epidermal growth factor (EGF)	R&D Systems	Cat# 236-EG
TrypLE	Gibco	Cat# 12604-013
DMEM/F12, no glutamine	Gibco	Cat# 21331-020
DMEM/F12, HEPES	Gibco	Cat# 31330-038
GlutaMAX	Gibco	Cat# 35050-038
Fetal bovine serum (FBS)	Gibco	Cat# 10270
mTeSR Plus	Stemcell Technologies	Cat# 05825
EDTA	Invitrogen	Cat# 15575020
Accutase	Thermo Fisher	Cat# A1110501
KnockOut Serum Replacement (KSR)	Gibco	Cat# 10828-028
MEM-NEAA	Gibco	Cat# 11140-035
ROCK inhibitor (Y-27632)	Sigma-Aldrich	Cat# Y0503-5 MG
Heparin	Sigma-Aldrich	Cat# H3149-10KU
Paraformaldehyde	Sigma-Aldrich	Cat# P6148
Activin A	PeproTech	Cat# 120-14E
Neurosphere Dissociation Kit	Miltenyi	Cat# 130-095-943

**Critical commercial assays**		

TaqMan Universal PCR Master Mix	Thermo Fisher	Cat# 4304437
High-Capacity cDNA Reverse Transcription Kit	Thermo Fisher	Cat# 4368814
TaqMan Assay – SOX17	Thermo Fisher	Hs007751752_s1, Cat# 4331182
TaqMan Assay – TUBB3	Thermo Fisher	Hs00742774_s1, Cat# 4331182
TaqMan Assay – GAPDH	Thermo Fisher	Hs02758991_g1, Cat# 4331182
RNeasy Mini Kit	Qiagen	Cat# 74104
Evercode Cell Fixation v2 Kit	Parse Biosciences	Cat# ECF2001/ECF2101
Evercode WT Mini v2 Kit	Parse Biosciences	Cat# ECW02010/ECW02110

**Deposited data**		

mRNA-seq data [https://www.omicsdi.org/dataset]: [GSE298718]	SCBT Research	https://www.ncbi.nlm.nih.gov/geo/query/acc.cgi?acc=GSE298718

**Experimental models: Cell lines**		

Human GBM1_FUCCI cells	SCBT Research	N/A
Human GBM4_FUCCI cells	SCBT Research	N/A
Human GBM20_FUCCI cells	SCBT Research	N/A
Human NP1_FUCCI cells	SCBT Research	N/A
Human GBM1_GFP cells	SCBT Research	N/A
HOIK_1 iPSC line	HipSci/Sanger	77650129

**Software and algorithms**		

Fiji *(ImageJ)*	Schindelin et al., 2012	https://imagej.nih.gov/ij/
BBrowserX	BioTuring	https://bioturing.com/
DyPheT	SCBT Research	https://github.com/kevcritc/DyPheT
BioRender	BioRender	https://www.biorender.com/
GraphPad Prism 10	GraphPad Software (V. 10.6.0)	https://www.graphpad.com
Inkscape	Inksacpe Project (V. 4.1)	https://inkscape.org
ZEISS Blue	ZEISS Blue Software (v3.11)	https://www.zeiss.com/microscopy/en/products/software/zeiss-zen.html?utm_source=chatgpt.com
Harmony	Revvity formerly Perkin Elmer Software (v.5.3)	https://www.revvity.com/gb-en/product/harmony-5-2-office-revvity-hh17000019

### Experimental model and study participant details

#### Human induced pluripotent stem cell (iPSC) culture

The Hoik_1 human iPSC line was maintained in mTeSR Plus medium (STEMCELL Technologies, Cat# 05825), a modified Tenneille Special Recipe formulation. Cells were cultured in a humidified incubator at 37 °C with 5% CO_2_ on vitronectin-coated flasks or plates (5 μg/mL; Gibco, Cat# A14700). For passaging, cells were dissociated using 0.5 mM EDTA (Invitrogen, Cat# 15575020).

#### Cell model (GB and NP) culture

Patient-derived GB stem-like cell lines (GBM1, GBM4, GBM20) were cultured^28^ as adherent monolayers on flasks coated with poly-L-ornithine (5 μg/mL; Sigma, P3655) and laminin (5 μg/mL; Invitrogen, 23017-015). For GBM1 and GBM4 model generation, tumor samples classified as primary GB were obtained with written informed consent from patients undergoing surgery at Stanford Medical Center under approval by the Institutional Review Boards of Stanford University and The Scripps Research Institute, USA (study approval numbers: ID76892 and 2006-HSC). The GBM20 model was derived from tumor tissue collected under informed consent and approval of the Leeds Multidisciplinary Research Tissue Bank, University of Leeds, UK (REC ref. 10/H1306/7). Cells were maintained in Neurobasal medium (Gibco, 21103-049) supplemented with B-27 (0.5×; Invitrogen, 17504-044), *N*-2 (0.5×; Invitrogen, 17502-048), recombinant human bFGF (40 ng/mL; Gibco, PHG0026), and EGF (40 ng/mL; R&D Systems, 236-EG) at 37 °C in a humidified 5% CO_2_ incubator. Cultures were passaged using TrypLE (Gibco, 12604-013). All GB lines were IDH-wildtype and selected to reflect clinically relevant tumor stages and previously published subtype characteristics.^28^ Briefly, GBM1 was derived from a 58-year-old female with a primary GBM and no prior therapy. The tumor exhibited MGMT promoter hypermethylation (67%), Chr7 amplification, and a mixed Classical/Proneural subtype profile. GBM4 originated from a primary tumor (patient age and gender not available) with no prior treatment, low MGMT promoter methylation (19%), Chr7 amplification, and a predominantly Mesenchymal profile with additional Classical/Proneural subtype features. GBM20 was established from a 50-year-old male with a recurrent GBM following radiotherapy, temozolomide, and IMA950 vaccination. The tumor showed unmethylated MGMT (2%), Chr7 amplification with Chr10 monosomy, and a mixed Proneural/Mesenchymal subtype signature. For live-cell imaging applications, cells were transduced with a lentiviral vector encoding TurboGFP under the control of the CMV promoter and a puromycin resistance cassette (MISSION pLKO.1-CMV-TurboGFP-Puro; Sigma-Aldrich, Cat# SHC003V). FUCCI-expressing derivatives were generated using the LTV-0052-1 vector system (LipExoGen) and selected using 5 μg/mL puromycin treatment.

Human neural progenitor cells (NP1) were derived from cortical tissue obtained during epilepsy surgery as previously described.^9^ Cells were maintained in DMEM/F12 (Gibco, 21331-020) supplemented with B-27 (0.5×), *N*-2 (0.5×), GlutaMAX (1×; Gibco, 35050-038), bFGF (20 ng/mL; Gibco, PHG0026), EGF (20 ng/mL), and 5% (v/v) fetal bovine serum (FBS; Gibco, 10270) at 37 °C with 5% CO_2_. Cells were passaged using TrypLE. NP1 cells were modified to express GFP via lentiviral transduction (MISSION pLKO.1-CMV-TurboGFP-Puro; Sigma-Aldrich, Cat# SHC003V), and Fluorescent Ubiquitination-based Cell Cycle Indicator^64^ (FUCCI)-labelled derivatives were generated (using the LTV-0052-1 vector system, LipExoGen) for cell cycle visualisation.

The cell models used in the study tested negative for mycoplasma. Cell models were not authenticated.

#### Human participants

Three human recurrent glioblastoma tissue sections from the tumor periphery were analyzed as one experimental group. They were obtained by neurosurgery at Leeds Teaching Hospitals under informed consent and approval by the Neuropathology Research Tissue Bank, University of Leeds, UK (REC ref. 2-YH-0109). The three participants included 1 male and 2 females, aged 45, 54 and 56 at diagnosis, and 46, 54 and 60 at recurrent surgery. The influence of sex, gender, or both on the results of the study are not known. All patients received chemoradiotherapy after initial surgical tumor debulking.

#### Generation of endodermal and neural linage GB ‘host’ environments

Human iPSCs were dissociated into a single-cell suspension via sequential incubation with 0.5 mM EDTA (Thermo Fisher, Cat# 15575020) followed by Accutase (Thermo Fisher, Cat# A1110501). Dissociated cells were seeded at a density of 4 × 10^5^ cells per well in vitronectin-coated (Thermo Fisher, Cat# A14700) 24-well plates, using mTeSR medium (STEMCELL Technologies, Cat# 100–0276) supplemented with 10 μM Y-27632 ROCK inhibitor (Sigma-Aldrich, Cat# Y0503). After 24 h, the medium was replaced with either endoderm or neural induction medium (STEMCELL Technologies, Cat# 05110 or 05839, respectively), and refreshed daily for an additional 4 days.

To initiate three-dimensional (3D) structure formation, induced cells were dissociated using the same EDTA/Accutase protocol and replated in ultra-low attachment 96-well plates (Corning) at a density of 4 × 10^4^ cells per well in 100 μL of their respective induction medium, supplemented with 50 μM Y-27632. After 24 h, 3D aggregates had formed, at which point 50 μL of medium was removed and replaced with 150 μL of maturation medium.

For neural organoid-like ‘hosts’, the maturation medium consisted of a 1:1 mixture of Neurobasal and DMEM/F12 (Thermo Fisher), supplemented with 1× B27 without vitamin A (Thermo Fisher), 1% N2 (Thermo Fisher), 1× GlutaMAX (Thermo Fisher), 0.5× MEM-NEAA (Thermo Fisher), 50 μM 2-mercaptoethanol (Thermo Fisher), and 25 μg/mL insulin (Sigma-Aldrich). For endodermal lineage (foregut) organoid-like ‘hosts’, the maturation medium comprised RPMI (Thermo Fisher) supplemented with 1× B27 (Thermo Fisher), 0.5× MEM-NEAA, and 50 ng/mL Activin A. Half-medium changes were performed daily for an additional 6 days. On day 7, matured ‘hosts’ were co-cultured with GFP-labelled GBM1 spheroids for invasion assays.

#### Generation of GB-FUCCI assembloids for cell cycle tracking

FUCCI-expressing, patient-derived glioblastoma lines (GBM1, GBM4, GBM20) and neural progenitor cells (NP1) [9, 24] were maintained as described under the Cell Model (GB and NP) culture section. Cerebral organoids were generated using a modified protocol based on.^55^ Hoik_1 iPSCs were dissociated using 0.5 mM EDTA followed by Accutase treatment (Thermo Fisher, A1110501), and seeded at 9000 cells per well in ultra-low attachment (ULA) 96-well plates (Corning, CLS7007) on Day 0.

Embryoid body (EB) formation was initiated in human stem cell (HSC) medium consisting of DMEM/F12 (Gibco, 31330-038), knockout serum replacement (20% v/v; Gibco, 10828-028), MEM-NEAA (1% v/v; Gibco, 11140-035), GlutaMAX (1% v/v), β-mercaptoethanol (55 μM), bFGF (4 ng/mL; Gibco, PHG0026), and Y-27632 (50 μM). Half-volume medium changes were performed on Days 2 and 4; Y-27632 was included only during the first four days. On Day 7, EBs were transferred to ULA 24-well plates (Corning, CLS3473) in neural induction (NI) medium comprising DMEM/F12, *N*-2 supplement (1% v/v), GlutaMAX (1% v/v), MEM-NEAA (1% v/v), and heparin (1 μg/mL). On Day 8, an equal volume of fresh NI medium was added.

In parallel, FUCCI-expressing GBM or NP1 lines were dissociated and seeded at 1000 cells per well into ULA 96-well plates, where they were cultured in their respective media to form spheroids over 24 h (37 °C, 5% CO_2_). On Day 9 of cerebral organoid development, organoids were transferred from ULA 24-well plates into the spheroid-containing ULA 96-well plates. This co-culture initiated assembloid formation, enabling subsequent GB invasion assays. Static live cell images (phase contrast and fluorescence) were acquired using an EVOS digital inverted fluorescence microscope (Life Technologies).

### Method details

#### Immunofluorescence and immunohistochemistry

Cells were fixed in 4% (w/v) paraformaldehyde (PFA; Sigma, P6148) prior to staining. Adherent cultures were fixed for 10 min at room temperature, then permeabilised with 0.5 M glycine in PBS containing 0.5% (v/v) Triton X-100 (Sigma, T8787). Organoids and assembloids were fixed in PFA for 15 min at 4 °C, washed three times in PBS, and transferred to 30% (w/v) sucrose solution until fully equilibrated. Sunk tissues were embedded in optimal cutting temperature compound (OCT; VWR, 36160E) using cryo-moulds and cryosectioned at ∼20 μm thickness.

Sections were blocked for 2 h at room temperature in staining buffer (PBS containing 10% (v/v) FBS and 0.04% (v/v) Triton X-100), then incubated overnight at 4 °C with primary antibodies diluted in the same buffer. The next day, sections were incubated for 1 h at room temperature with fluorophore-conjugated secondary antibodies, counterstained with 1 μM DAPI (Sigma), and imaged using an EVOS inverted fluorescence microscope. To assess the proliferative potential of human GB cells in peripheral tissue post-surgery, we performed MKI67 staining (1:200 primary antibody dilution) on tissue sections as described above. Stained sections were imaged at 20× magnification using the Axion Scan.Z1 slide scanner, and images were analyzed with ZEISS Blue software (v3.11). MKI67-positive nuclei were manually quantified based on DAPI co-staining. Biological replicates correspond to individual glioblastoma patients, while technical replicates represent equally sized regions within the margin zone of each tissue sample. On average, 651 cells were analyzed per patient.

#### Quantitative real-time PCR (qRT-PCR)

Total RNA was extracted from assembloid samples using the RNeasy Mini Kit (Qiagen, Cat# 74104), following the manufacturer’s protocol. RNA concentration and purity were assessed using a NanoDrop spectrophotometer (Thermo Fisher Scientific). Complementary DNA (cDNA) was synthesised from 1 μg of RNA using the High-Capacity cDNA Reverse Transcription Kit (Thermo Fisher Scientific, Cat# 4368814).

Quantitative real-time PCR was performed using TaqMan Gene Expression Assays (Thermo Fisher Scientific) and TaqMan Universal PCR Master Mix (Cat# 4304437) on a QuantStudio 5 Real-Time PCR System (Applied Biosystems). The following TaqMan assays were used: SOX17 (Assay ID H00751752_s1, Cat# 4331182) as an endoderm lineage marker, and TUBB3 (Assay ID, Hs00742774_g1) as a neural lineage marker. GAPDH (Assay ID Hs02758991_g1) was used as the endogenous control.

Each 20 μL reaction consisted of 10 μL master mix, 1 μL assay mix, 2 μL cDNA, and 7 μL nuclease-free water. Thermal cycling conditions were 50 °C for 2 min, 95 °C for 10 min, followed by 40 cycles of 95 °C for 15 s and 60 °C for 1 min. Reactions were performed in technical triplicates. Relative expression was calculated using the 2ˆ–ΔΔCt method, comparing gene expression in neural GB assembloids versus endodermal GB assembloids, and results are reported as fold change.

#### Single-cell RNA sequencing analysis

After 48 h of the GB invasion protocol, assembloids were dissociated in microcentrifuge tubes using the Neurosphere Dissociation Kit (Miltenyi, 130-095-943) according to the manufacturer’s instructions. The resulting single-cell suspension was passed through a 70 μm cell strainer, followed by centrifugation at 300*g* for 5 min. The pellet was resuspended in 500 μL of PBS containing 2 μM EDTA, 5% Bovine Serum Albumin (Merck, A4503), and 1× Penicillin-Streptomycin (Merck, P0781). Glioblastoma (GB) cells and host cells were separated based on GFP expression using a BD Influx 6-Way Cell Sorter. Sorted cells were pooled at a 1:1 ratio and processed for single-cell RNA sequencing. Library preparation was performed using the 10x Genomics GEM-X 3′ v4 assay, and sequencing was carried out on a NovaSeq X Plus Instrument (10B, PE50).

Single-cell RNA sequencing (scRNA-seq) libraries were pre-processed to retain cells with more than 5,000 unique molecular identifiers (UMIs). Gene expression counts were log-normalized using a scale factor of 10,000. Dimensionality reduction was performed via principal component analysis (PCA) utilising 33 principal components. Cell clustering was conducted using the Louvain algorithm on a k-nearest neighbor graph (k = 100, resolution = 0.3), identifying 17,424 high-quality cells. Non-linear dimensionality reduction visualisations, including UMAP and t-SNE, were generated using BioTuring BBrowserX^65^ and BioVinci (v3.5.26).

#### Differential gene expression analysis

Differential gene expression analysis was performed in BioTuring BBrowserX^65^ using the non-parametric Wilcoxon rank-sum test to compare GB control cells with GB cells invading neural, non-neural, and combined environments. Genes were filtered using an adjusted *p*-value threshold of <0.01 and log2 fold change (Log2FC) > 1 or < −1, resulting in 553, 456, and 776 differentially expressed genes for the combined, neural, and non-neural invading conditions, respectively. Overlapping genes common to all three conditions were identified as the intrinsic GB invasion signature (IGI-S), while genes unique to the neural and non-neural environments were characterized as the ‘extrinsic’ gene signatures for these respective conditions.

#### Functional enrichment and network analysis

Gene set enrichment analysis was performed using Gene Ontology Biological Processes gene sets obtained from the Molecular Signatures Database (MSigDB) (https://www.gsea-msigdb.org/gsea/msigdb). Analysis was conducted using the ClusterProfiler^32^ R package, applying a significance threshold of *p* < 0.05. For protein–protein interaction (PPI) network construction, differentially expressed genes were submitted to the STRING^35^ database (v11.5), using Homo sapiens as the reference organism and a minimum interaction confidence score of 0.7. The resulting high-confidence interaction network was imported into Cytoscape^66^ (v3.10.12) for visualisation, and unconnected nodes were removed. Hub gene analysis was carried out using the CytoHubba^36^ plugin, applying the bottleneck topological method to identify the top 10 hub genes. The resulting 10-gene hub signature was validated against the dataset from Neftel et al.^33^ using the BioTuring Talk2Data database.

#### Survival analysis and data visualisation

Overall survival analysis was performed on TCGA-GBM patient samples using the survminer R package employing the log rank test. Kaplan–Meier curves were generated for the 10-gene hub signature, 59 upregulated IGI-S genes, and 86 downregulated IGI-S genes. Volcano plots were created using ggplot2 to visualise differential expression between conditions (*p* < 0.05, Log2FC > 1 or < −1), with the top significant genes labeled. Analysis of gene expression in neural and non-neural environments was carried out using the Seurat R package^67^ and cell type annotation using the SingleR R package and the Human Primary Cell Atlas reference dataset.^31^

#### Characterisation of GB-FUCCI workhorse model by scRNAseq

GB-FUCCI ‘workhorse’ cells were derived from the patient-derived GBM1 model^37^ and enzymatically dissociated from adherent cultures using TrypLE Express Enzyme (Thermo Fisher Scientific, Cat# 12604013). Cell suspensions were centrifuged at 300 × g and resuspended in NB medium. Samples were fixed using the Evercode Cell Fixation v2 kit (Parse Biosciences, ECF2001/ECF2101) following the manufacturer’s instructions. Fixed cells were stored at −80 °C for up to 1 month prior to barcoding. Library preparation was performed according to manufacturer instructions using the Evercode WT Mini v2 kit (Parse Biosciences, ECW02010/ECW02110). In brief, fixed single-cell suspensions underwent a series of intracellular, instrument-free reverse transcription barcoding reactions. Resulting barcoded libraries were divided into sub-libraries as required, with an additional sub-library-specific barcode introduced. Final sub-libraries were lysed, and barcoded cDNA was amplified by PCR and prepared for Illumina sequencing. Sub-library quality control was carried out using a Tapestation 4200 (Agilent). Sequencing was performed using the NovaSeq 6000 platform with a resultant 100% clean reads.

For bioinformatic analysis, FASTQ files were delivered via the sequencing provider (Novogene) cloud-based platform. Following data integrity verification using a Message-digest algorithm 5 (MD5) checker to ensure complete cloud delivery, sequencing data were concatenated and aligned to the human genome reference GRCh38 (HG38) using the PARSE bioinformatics pipeline. The resulting output was formatted for compatibility with 10x Genomics analytical workflows and analyzed using the BioTuring ‘BBrowser’ platform (https://bioturing.com/). Cells with less than 10 reads or 10 gene counts were excluded. After filtering, a total of 5,938 cells were retained, with a median of 4,014 transcripts and 2,352 genes detected per cell, with a median mitochondrial gene percentage of 0.04%. Expression of selected genes was visualised using UMAP plots generated via the platform’s default input mask. Using published gene sets, the AUCell enrichment function within BBrowser was employed to assign individual cell scores based on predefined gene signatures, including G1/S, G2/M, astrocyte-like (AC-like), mesenchymal-like (MES-like), neural progenitor-like (NPC-like), and oligodendrocyte progenitor-like (OPC-like) states. These cell-wise scores were then used to adapt the butterfly graph technique originally described by Neftel and colleagues.^33^ Briefly, each cell was assigned a value “D”, calculated as: D = MAX (OPC-like score, NPC-like score) – MAX (AC-like score, MES-like score), Y axis values were derived using: Y = Log_2_ (D + 1); X axis values were computed as follows: If D > 0: X = Log_2_ [(OPC-like score – NPC-like score) + 1], If D < 0: X = Log_2_ [(AC-like score – MES-like score) + 1]. Additionally, a cell cycle score (CCS) was calculated for each cell as a measure of proliferative activity: CCS = G1/S score + G2/M score.

#### Time-lapse confocal microscopy

Time-lapse live-cell imaging was performed on the Opera Phenix High-Content Screening System (PerkinElmer, Waltham, MA), which features a confocal spinning disk system with four solid-state lasers (405 nm, 488 nm, 561 nm, 640 nm; each 50 mW) and a fixed light path configuration. Emission filters used included 435–550 nm, 435–480 nm, 500–550 nm, 570–630 nm, and 650–760 nm.

Image acquisition was performed with four dedicated Zyla sCMOS cameras (Andor, Belfast, UK), each with 2160 × 2160 (pixel) resolution and 6.5 μm pixel size. A single central field of view was imaged per well with a z stack range of 200 μm, acquired in 4 μm steps, over approximately 65 hourly time points. All imaging was carried out at 37 °C in a humidified chamber with 5% CO_2_ using an Olympus 10× air objective (NA 0.3, WD 5.2 mm). Exposure times were 20 ms at 50% laser power for Brightfield and 561nm^ex^/570 nm-630^em^, and 200 ms at 100% power for 488^ex^/550 nm-550nm^em^ channels. Data acquisition was controlled using Harmony software (Revvity formerly PerkinElmer) and archived for storage.

#### Image and video analysis

Analysis of static fluorescence and phase-contrast images acquired from EVOS and Opera Phenix systems was performed using Fiji.^68^ Real-time 2D and 3D time-lapse videos were analyzed using a bespoke algorithm, DyPheT (see dedicated methods section). Data were collated in Microsoft Excel 2019 and subjected to statistical analysis using GraphPad Prism (version 10).

Quantification of assembloid invasion was performed in Fiji. Fluorescent images acquired at 0, 24, and 48 h were converted to grayscale, and the thresholding function was applied to selectively gate the FUCCI-GB fluorescence signal while excluding background fluorescence and non-invading cells. Manual measurements were taken for both the total organoid area and the area of internalised fluorescence at each timepoint. These measurements were used to calculate the percentage of GB cell infiltration per assembloid over time. Statistical comparisons were made using two-way repeated measures ANOVA, followed by uncorrected Fisher’s least significant difference (LSD) test for post hoc analysis.

#### DyPheT development and utilisation

The DyPheT algorithm was developed in Python 3.10.9. The algorithm primarily uses several methods from the OpenCV library to perform registration between the video frames, to automatically rotate and translate video frames, ensuring that the assembloid (or other 3D organoid/spheroid structure) remains centered and correctly orientated throughout the time-lapse sequence. This enables accurate tracking of individual cell movements relative to a fixed reference point.

The center of the assembloid was defined as the center of mass of its outline in the first frame. The outline was extracted by applying a sequence of image-processing steps: filtering, thresholding, contouring, and convex hull computation. The calculated center of mass was translated to the center of the image frame to serve as the reference point for motion tracking. To align subsequent frames (n and n+1), template matching was applied using OpenCV’s TM_CCOEFF_NORMED method with a minimum of six templates selected from within the assembloid region. This allowed positional adjustment in the x- and y axes to re-centre the assembloid in frame n+1. The same frames were then subjected to polar transformation using a warp function centered on the organoid, creating polar-warped versions of both frames.

Rotation between frames was determined by template matching (TM_SQDIFF_NORMED) up to 40 defined regions across the polar-warped images. The detected angle of rotation, θ, was applied to frame n+1. Following this, angular correction, x- and y axis alignment was repeated, followed by another round of polar-based rotation estimation using the updated center point. This translation–rotation process was iteratively refined (typically 4–12 iterations per frame pair), stopping when either a convergence threshold was reached, or the maximum number of iterations (typically 15) was completed. Smoothing was applied to minimise oscillations during the process.

Throughout the analysis, the convex hull of the assembloid served as a mask to ensure template matching was restricted to the biological structure, minimising background interference. All images were converted to grayscale for analysis. The uncertainty in θ between frames was typically less than 0.5°.

DyPheT-generated data included positional coordinates and motility metrics of individual cells, as well as detected hue values per frame. Output plots included vector maps and invasion heatmaps. All raw data were exported as Excel worksheets, enabling post hoc analysis such as spatiotemporal tracking of individual or grouped cells within an assembloid and testing for statistical differences across various conditions.

#### Compound treatment

For the proof-of-concept compound trial, R-P6306 (Cambridge Bioscience, Cat# HY-145817A-5 mg) was prepared as a 10 mM stock solution in DMSO. Serial dilutions were used to achieve a final working concentration of 50 nM. Control assembloids were treated with an equivalent DMSO concentration (0.0005%). The assembloid invasion assay was conducted exactly as described above, with compound or vehicle treatment applied throughout the co-culture period.

### Quantification and statistical analysis

All statistical analyses were performed using GraphPad Prism (version 10) and relevant R packages for single-cell and survival analyses. Biological replicates (≥3) were used for all experiments, and technical replicates were included for qRT-PCR quantifications. Normality was assessed using the Shapiro–Wilk test. Where data followed a normal distribution, unpaired two-tailed Student’s t-tests were used for two-group comparisons. Fisher’s Least Significant Difference (LSD) test was applied post hoc to identify pairwise differences. For non-normally distributed data, the Mann–Whitney U test was used.

For single-cell RNA-seq differential gene expr*ession, the Wilcoxo*n rank-sum test was performed in BioTuring BBrowserX, with Benjamini–Hochberg false discovery rate (FDR) correction applied. Adjusted *p*-values <0.01 were considered significant.

For overall survival analysis, Kaplan–Meier curves were generated, and group differences were assessed using the log rank test via the survminer R package. A significance threshold of *p* < 0.05 was applied throughout results.
