# Supplementary material for: Glioblastoma invasion into different organoid hosts reveals cell-intrinsic and proliferative migratory programs
Source: iScience. 2026 Mar 13;29(4):115361. doi: 10.1016/j.isci.2026.115361 (PMC13059114; doi:10.1016/j.isci.2026.115361)

iScience, Volume *29*

Supplemental information

Glioblastoma invasion into different organoid hosts reveals cell-intrinsic and proliferative migratory programs

Christopher Y. Akhunbay-Fudge, Bronwyn K. Irving, Alima Ismail, Sabrina Samuel, Emma Smedley, Holly E. Bradford, Steven Bagley, Alexander Baker, Iain M. Hagan, Deena M.A. Gendoo, Kevin Critchley, Ryan K. Mathew, and Heiko Wurdak

SUPPLEMENTAL INFORMATION

1. Supplementary Figures – pages 2-9
2. Methods S1 – Image Analysis Pipeline – pages 10-14

1. Supplementary Figures

Figure S1

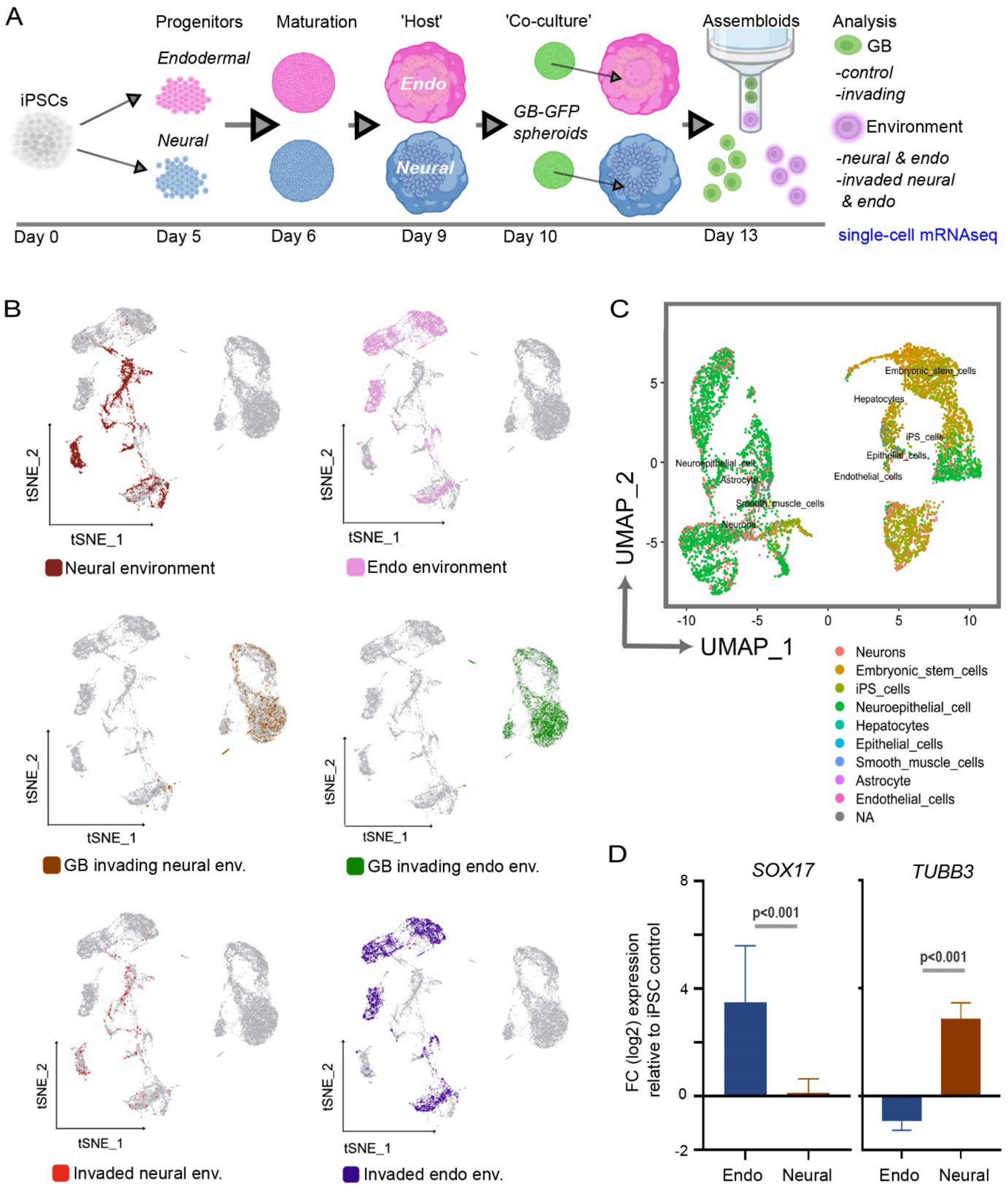

Figure S1. [Characterisation of neural and endodermal environments in GB assembloids], Related to Figure 1. (A) Schematic of the GB assembloid formation protocol. Human iPSCs were differentiated into neural and endodermal progenitors (Day 5), then matured to form

respective spheroids (Day 6–9). On Day 10, GB-GFP spheroids were co-cultured with neural or endodermal 'hosts' to form assembloids. At Day 13, assembloids were dissociated into single cells and subject to FACS before samples were processed for single-cell mRNA sequencing, enabling analysis of GB cells (control vs invading) and their corresponding environments. (B) UMAP plots showing neural and endothelial environment cells, GBM cells invading each environment, and the corresponding invaded cells, highlighting distinct invasion-associated (scRNAseq) clustering patterns. (C) UMAP plot showing all cell types identified across samples, annotated using.[31] Key environment-related cell types include neuroepithelial cells, neurons, smooth muscle cells, hepatocytes, and endothelial cells. (D) qRT-PCR analysis of environment-specific markers. SOX17 (endodermal marker) and TUBB3 (neural marker) are differentially expressed between endodermal and neural assembloid conditions. Data are shown as fold change ($\log_2$) relative to iPSC control; error bars represent standard deviations from the mean.

Figure S2

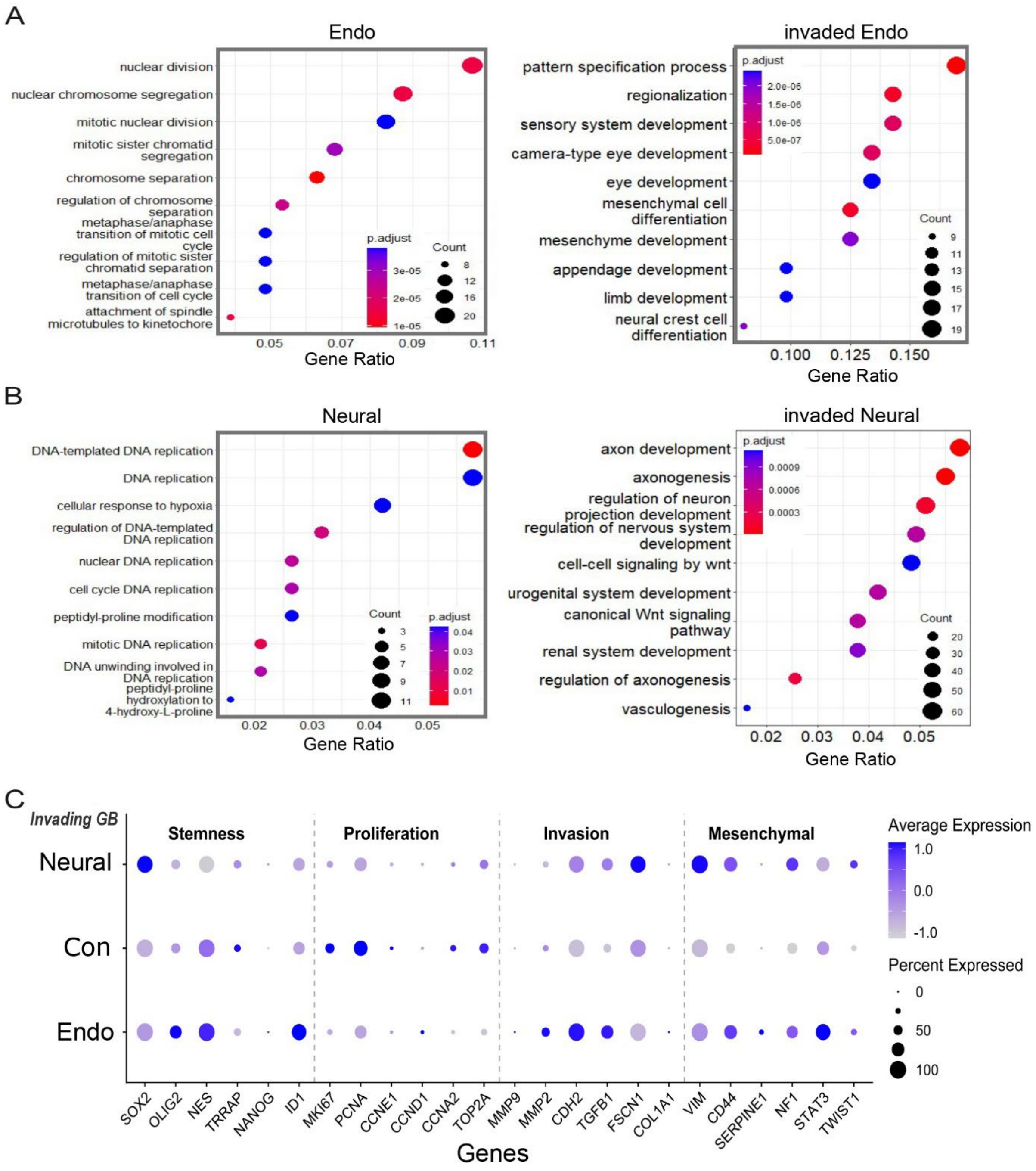

Figure S2. [Functional enrichment and marker expression in GB cells invading endodermal and neural environments], Related to Figure 2. (A) Gene Ontology (GO) Biological Process enrichment analysis of the endodermal extrinsic signature: genes uniquely expressed in the endodermal environment during GB invasion (left), and genes differentially expressed between uninvaded and GB-invaded endodermal environments (right). (B) GO enrichment analysis of the neural extrinsic signature: genes uniquely expressed in the neural environment during GB invasion (left), and genes differentially expressed between uninvaded and GB-invaded neural environments (right). Adjusted p-values < 0.05. (C) Bubble plot showing expression patterns of genes associated with stemness, proliferation, invasion, and

mesenchymal programmes across GB cells invading endodermal and neural environments compared to baseline control. Bubble size indicates the percentage of expressing cells; colour denotes average expression level.

Figure S3

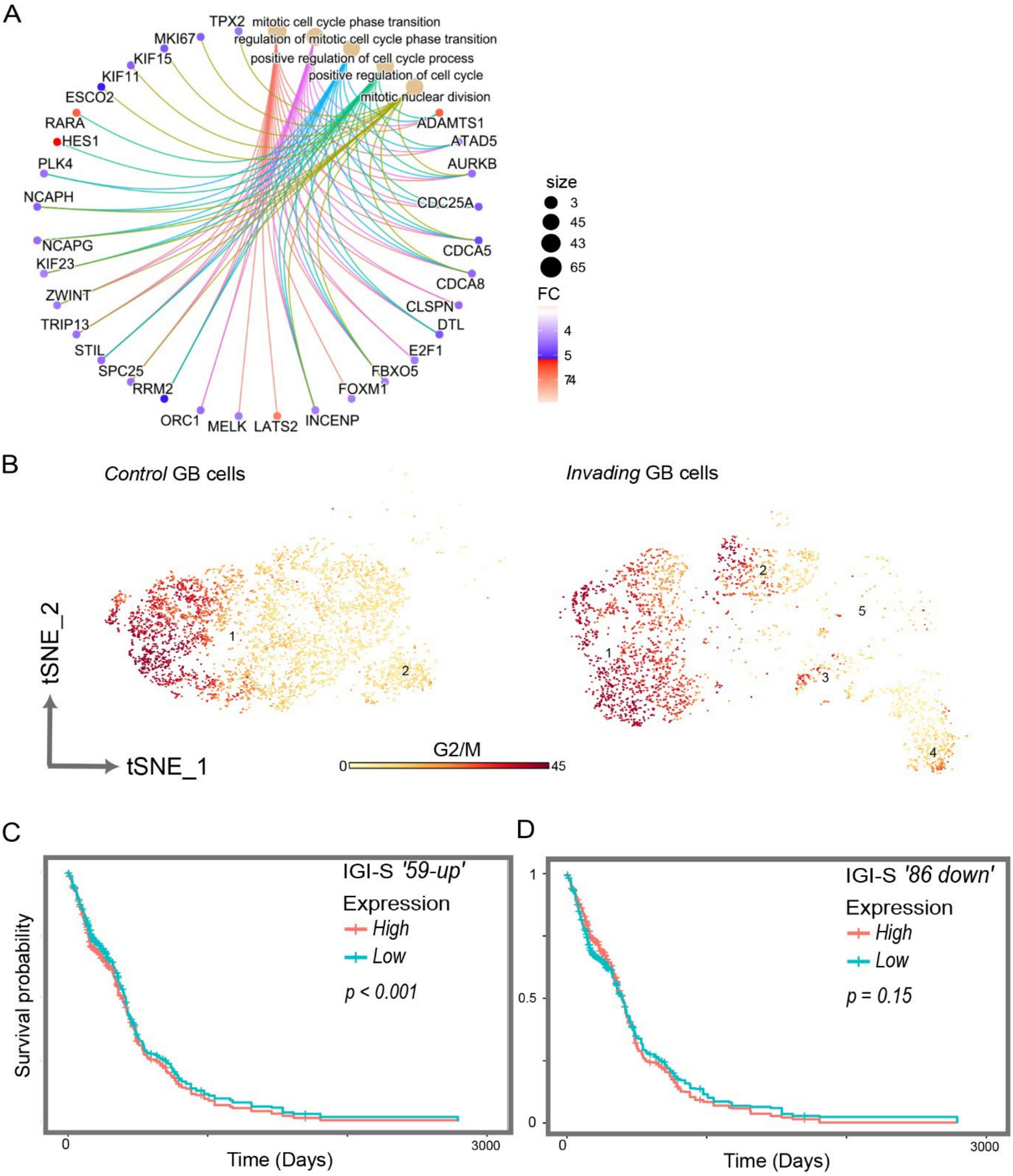

Figure S3. [Functional connectivity and clinical relevance of IGI-S], Related to Figure 3. (A) Cnetplot showing genes from the IGI-S present in the top five enriched Gene Ontology (GO) Biological Process pathways. Node size represents the number of genes per pathway; edge colour reflects fold change (FC). (B) t-SNE projection of single GB cells showing relative expression of the Neftel et al. G2/M gene set (scaled 0–45).[33] Left, control GBM1 cells; right, GBM1 cells invading the neural and non-neural host environments in 3D co-culture. A preserved G2/M-high population is maintained during invasion, with invading cells distributing into a broader set of subclusters (1–5) emerging under assembloid (co-culture) conditions. (C and D) Kaplan–Meier overall survival analysis of TCGA GB patients stratified by high vs. low expression of upregulated (C) and downregulated (D) IGI-S genes. Log-rank test significance as indicated.

Figure S4

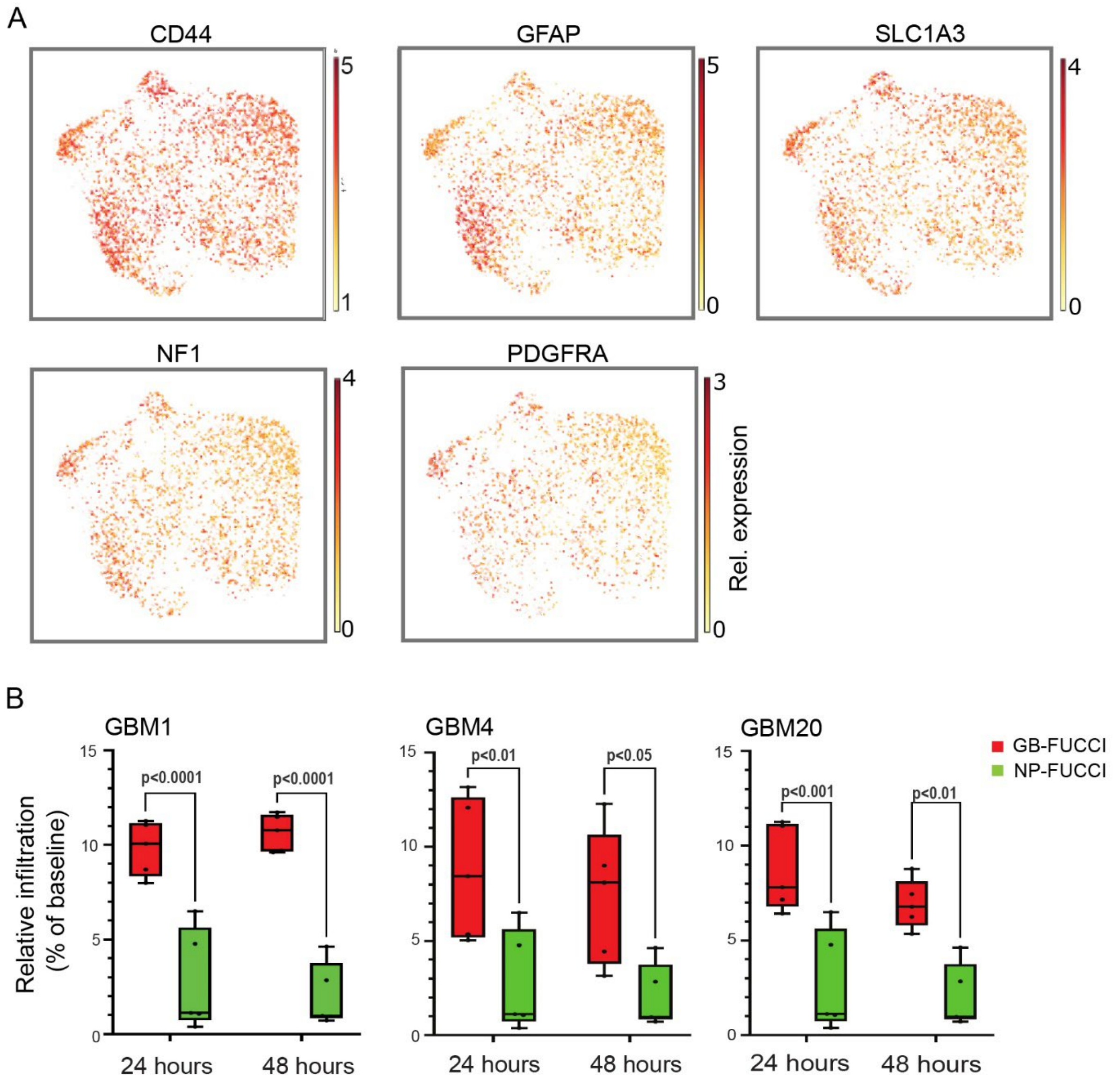

Figure S4. [GB-FUCCI subtype marker expression and cell cycle–dependent infiltration dynamics in assembloids], Related to Figure 4. (A) UMAP visualisation of single-cell transcriptomes from GB-FUCCI cells (GBM1 line), showing expression of glioblastoma subtype-associated genes: CD44 (mesenchymal), GFAP and SLC1A3 (astrocytic), NF1 (mesenchymal/proneural), and PDGFRA (proneural). (B) Quantification of relative infiltration (% of baseline; error bars represent standard deviations) in assembloid invasion assays using three patient-derived GBM lines (GBM1, GBM4, GBM20) expressing FUCCI. GB-FUCCI cells (red) exhibited significantly greater infiltration at 24 and 48 hours compared to non-proliferating FUCCI controls (NP-FUCCI, green. Box plots show the 25th–75th percentiles; the centre line indicates the median; whiskers depict the minimum and maximum values. Dots represent individual assembloids (n = 5). Statistical significance was determined using two-way repeated measures ANOVA, followed by uncorrected Fisher's least significant difference (LSD) test for post hoc comparisons.

Figure S5

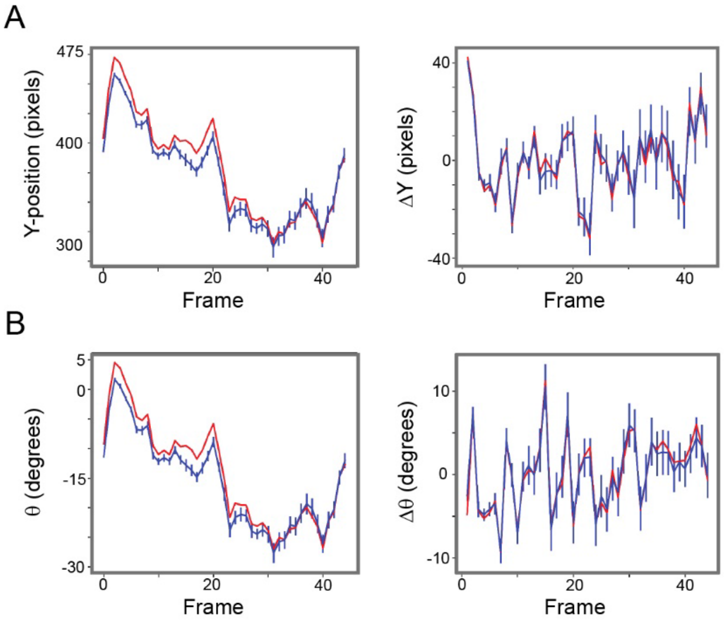

Figure S5. [Frame-to-frame correction of assembloid positional drift using DyPheT], Related to Figure 5. (A) Left: Y-position over time (frame number) for one selected assembloid invasion video. The red line shows automated correction using DyPheT; the blue line shows the average of manual annotations from three independent validators with standard error bars. Right: frame-to-frame change in Y-position (ΔY), comparing manual (blue) and automated (red) corrections. (B) Left: Rotation angle (θ) over time for the same video, with DyPheT and manual values as in (A). Right: frame-to-frame change in rotation angle (Δθ), showing strong agreement between manual and automated alignment.

Figure S6

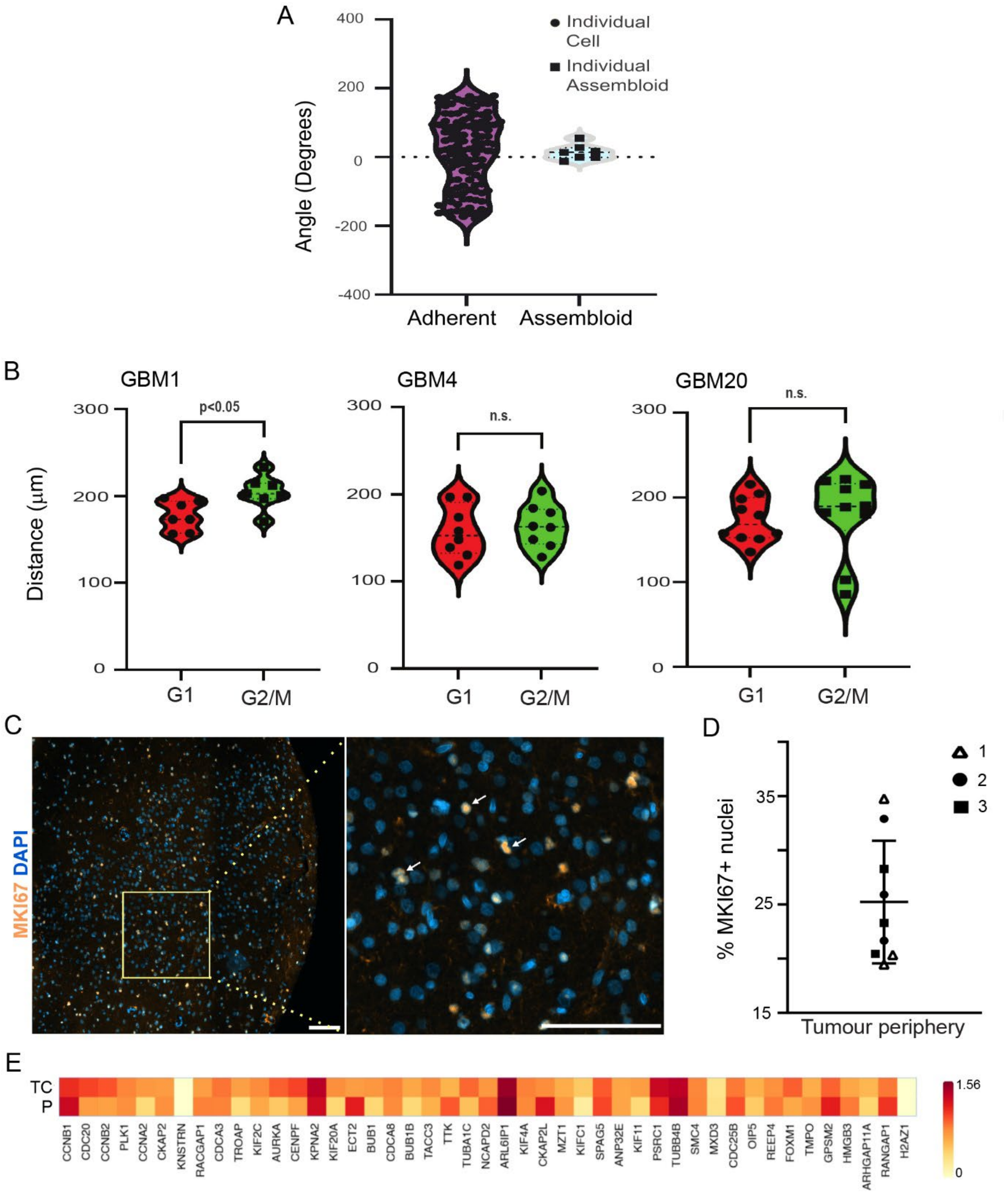

Figure S6. [Migration dynamics and cell cycle–dependent motility of GB-FUCCI cells in assembloids], Related to Figure 6. (A) Violin plots showing the distribution of migratory angles for GB-FUCCI cells cultured under adherent (2D) or assembloid (3D) conditions. Individual data points represent either single-cell trajectories (dots) or mean values per assembloid (squares). Cells in adherent cultures show broader directional variability compared to those in assembloids. (B) Violin plots comparing migration distances of G1-phase (red) versus G2/M-phase (green) GB-FUCCI cells in assembloids derived from three GB models (GBM1, GBM4, GBM20). GBM1 shows increased motility in G2/M cells, while GBM4 and GBM20 exhibit no significant differences. Data points represent mean values per assembloid. Statistical

comparisons were performed using unpaired two-tailed Student's t-tests. (C) Patient tumour-periphery tissue obtained from surgical resection was sectioned and stained for DAPI (nuclei) and MKI67 (proliferation marker). Left, lower magnification overview of the tumour periphery; right, higher-magnification view of the boxed region; MKI67-positive nuclei are indicated (arrows), scale bars 100 mm. (D) MKI67-positive nuclei as percentage of total (DAPI) nuclei; >500 nuclei were analysed per tumour imaging field across three fields (n = 3 patients). (E) Heatmap of log-normalised expression of Neftel et al. G2/M gene set[33] in single-cell RNA-seq data from Darmanis et al.,[40] comparing single cell gene expression from tumour core (TC) and periphery (P) regions. The G2/M proliferation signature is preserved in peripheral tumour cells.

Figure S7

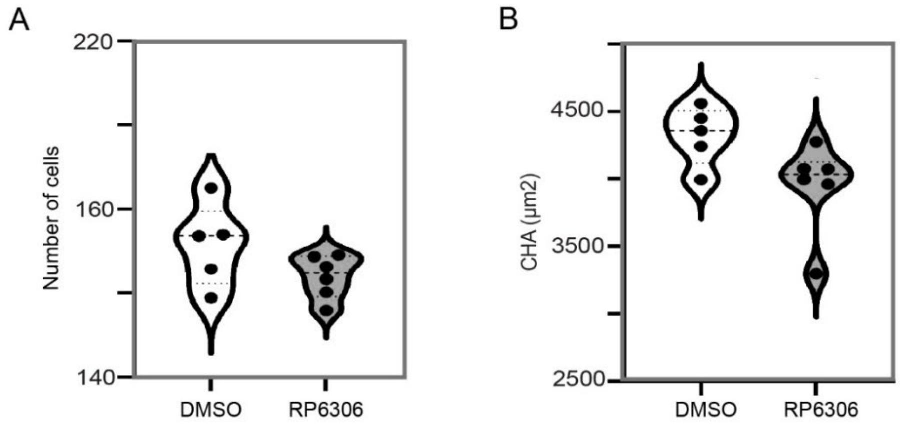

Figure S7. [DyPheT quantification of RP-6306-induced changes in GB-FUCCI cell dynamics], Related to Figure 7. (A) Violin plot showing the number of GB-FUCCI cells per assembloid following treatment with RP-6306 (50 nM) or DMSO control (0.0005%). No significant difference in total cell count was observed between treatment groups. (B) Violin plot showing convex hull area (CHA) values in GB-FUCCI assembloids treated with DMSO or RP-6306. Each dot represents an individual assembloid; horizontal lines within the violins denote the median and interquartile range. Sample sizes: DMSO (n = 5), RP-6306 (n = 6).

2. Methods S1 – Image Analysis Pipeline

2.1 Overview

The image analysis pipeline operates in two main stages: (1) motion correction of the assembloid using brightfield and fluorescent overlay videos, and (2) fluorescence-based cell detection and tracking. The code is designed to handle significant growth and movement of the assembloid over time, which poses challenges for conventional motion tracking algorithms. As humans we tend to focus a clear feature and match to that, but this can introduce drift if for example one side is the focus and the matching, over the other. The algorithm aims to create a consistent method to solve this problem.

2.2 Assembloid motion correction

To correct for translation and rotation of the assembloid across frames, the method aligns each frame n with frame n+1 using a combination of image preprocessing, template matching, and iterative refinement. Since frames n and n+1 are different sampling several areas was important, but to do this a reasonable estimate for the centre of the assembloid is needed to allow template matching algorithms to perform well.

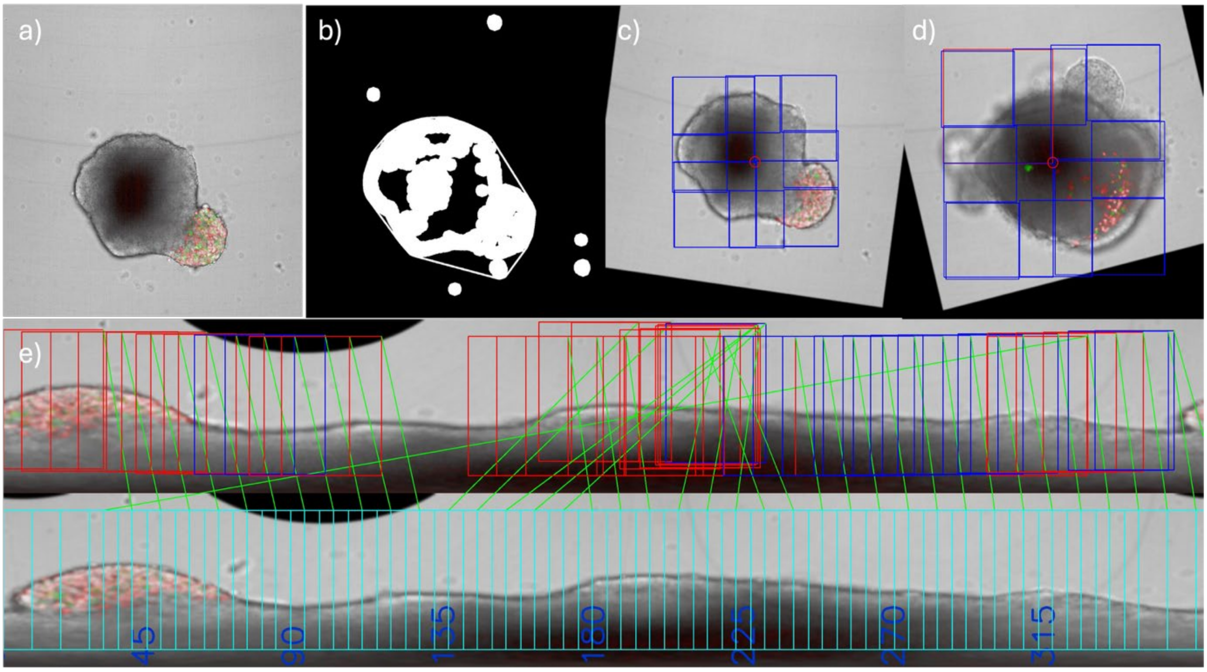

Examples (above) are shown for: a) a typical unaltered overlay frame n. b) a highly-distorted adaptive threshold inverted image after using the contour detection to determine the convex hull area for centre of mass estimate. c) frames 1 and 2 overlayed, with the 1 being rotated and translated by template matching panels after two iterations. The fits are for both

2 ´ 2 and 3 ´ 3 combined. d) frames 45 and 46 being matched by the same method, note that the assembloid has changed considerably. e) typical polar warp images for frames 1 (bottom) and 2 (top) with the respective template matches indicated by green connecting lines, in this instance 30 regions are being sampled. The red panel is match that is below a confidence threshold. The rotation can be quantified by the shift to the left. The blue numbers are the absolute angles of the image, and the height is the radius from the centre.

2.2.1 Preprocessing and centring

Each frame was converted to grayscale and processed using adaptive inverted thresholding. Morphological closing and dilation are applied to enhance the assembloid structure. Contours are extracted and filtered by size; the largest contour is taken to represent the assembloid. On the first frame, the convex hull of the contour is used to compute the centre of mass, which is used to translate the assembloid to determine centre of the frame and set the centre point for the polar warp. This method is repeated on subsequent frames however it becomes a first estimate of the central position and is fine-tuned by addition translation estimates. Performing the fine tuning without this initial step can lead to no matches being returned between frames, i.e. the method requires an estimate to being the registration.

2.2.2 Translation estimation

For frames n > 1, the frame is initially rotated by the previously estimated rotation angle (e.g. 0° for the second frame). A grid of templates (e.g. 2×2) from frame n is matched to frame n+1 using OpenCV template matching using the TM_CCOEFF_NORMED method to allow masking. In the cases where too few quality matches are made the method is switched to another method and this can help provide fits in these rare situations and allow the algorithm to proceed without failure. Without a reasonable rotation estimate the template matching will perform poorly however there are some conditions to prevent unstable and unlikely shifts in centre position. Further, to increase accuracy eight panels from a 3×3 grid (missing the centre) is matched in addition to the 4 panels from the 2×2 grid. The median shift in x and y across all templates is used to adjust the translation. The same matching between frames n and n+1 continues with each pair. Panel d (page 9) shows the penultimate and final frames being matched. The separate panels give some freedom for growth of the assembloid without biasing a shift along a particular axis. The required shifts to the centre position are damped to reduce oscillatory behaviour, based on the standard deviation of the range of detected matches.

2.2.3 Rotation estimation

A polar transformation was applied to the grayscale image on which has been translated to the estimated centre. Template matching is performed in polar coordinates to estimate the rotation angle. Many matching templates are created from the previous polar image and are matched to the current frame. For some assembloids it is better to sample fewer, but larger matching windows and vice versa – some manual optimisation can improve registration results. The templates are also masked using a polar transformation of the filled hull, so that the matching does not include the area outside the assembloid. An OpenCV ORB key point detector can be used to identify feature points; however, this approach was found to be ineffective for the types of features present in these videos. In practice, uniform angular sampling outperformed just as well and is more predictable. Typically, between 18 to 36 template windows, spaced evenly, of a width of 300 pixels performed well. The match angular positions are filtered to remove outliers. The key point detection can be selected to run at regular intervals during the alignment.

The algorithm iteratively alternates between translation and rotation adjustments until they converge. Both linear and angular transformations are applied using OpenCV's warpAffine function, which enables accurate alignment of frames. The angular rotation needs a good estimate of the centre, and the centre alignment needs a reasonable angular rotation estimate - they are not entirely independent measurements, although in most of the cases convergence to a good overlay position is quite rapid. For some frames the alignment alternates between poor and good due to the translation and then rotation. Some 'skipping' logic has been added to detect and solve this by skipping the rotation change for two or three rounds. To reduce drift, there is also a check against the current frame, n, and n-2. This reduces the speed of the analysis. It is simple to adapt the code to match even older frames, but the caveats are the matching confidence between larger time gaps decreases and the computational time needed. An average composite of all frames (a) is shown below (see page 12) along with a maximum intensity projection (b) of a centre tracked assembloid. The growth of the assembloid size over the experiment is significant but the algorithm provides excellent tracking for rotation and position. If there are many poor matches during the alignment of two frames then there is a reset of the last good fit, along with an increasing the angular sampling templates.

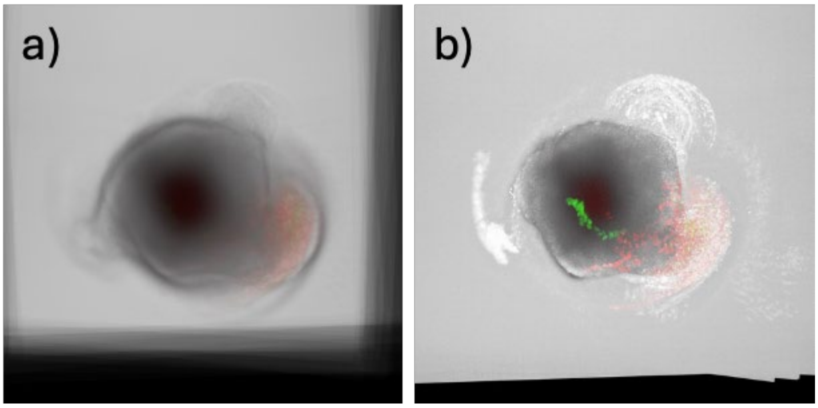

2.2.4 Robustness and limitations

Conditional checks are included to discard implausible rotation estimates (e.g., reject >60°), though this angle threshold may need to be adapted for each video if large rotations between frames occur. There are fall-back logic methods that prevent the alignment being trapped in a local minimum or to skip the rotation for a set number of rounds during the alignment, when it has been established that the rotation angle is close to a good fit. If the assembloid moves out of the field of view or becomes partially obscured, the matching may fail, requiring manual correction. Manual correction can be enabled in the code and the use of keys to align the frames is performed by hand. This was used to validate the automated method, and all other data was processed by the automated method. Frame overlays are generated during processing to allow visual inspection of alignment quality. These include frame n and n+1 rotated and translated, as well as frame n and frame 1.

2.3 Fluorescence image analysis and cell tracking

Once assembloid motion correction is complete, the recorded translation and rotation matrices were applied to the fluorescence video for cell detection and tracking.

2.3.1 Detecting cells

Fluorescence frames are processed using hue-based colour filtering, grayscale conversion, background subtraction, and smoothing. Morphological opening with an elliptical kernel is applied, followed by thresholding and contour detection. Contours are filtered by size and circularity to identify cell-like objects. For each detected cell, the following features are extracted: position, area, mean hue, intensity, and a unique identifier.

2.3.2 Cell tracking

A nearest-neighbour algorithm is used to match cells between consecutive frames based on spatial proximity and colour similarity. This enables the construction of trajectories for individual cells over time. The example diagrams below show a carton of the nearest-neighbour pairing method. The distance between each cell in frame n is measured to each cell in frame n+1. An array is created to store this information. The nearest cells between the frames are pair provided they are within an allowed distance (dashed circles) and are within a tolerance of the change in hue.

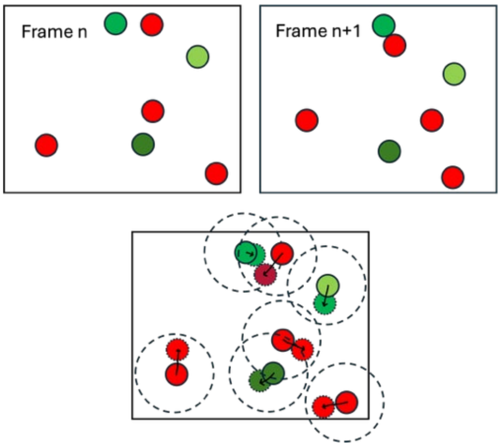

Supplement: Document S1. Figures S1–S8 and Methods S1 [file mmc1.pdf]
